# Myelin basic protein enhances axonal regeneration from neural progenitor cells

**DOI:** 10.1186/s13578-021-00584-7

**Published:** 2021-04-29

**Authors:** Zhengjian Yan, Lei Chu, Xiaojiong Jia, Lu Lin, Si Cheng

**Affiliations:** 1 Department of Orthopedics, the Second Affiliated Hospital of Chongqing Medical University, No. 76 Linjiang Road, Yuzhong District, Chongqing, 400010 China; 2 Department of Orthopedics, the First Affiliated Hospital of Chongqing Medical University, Chongqing, China

**Keywords:** Spinal cord injury, Axonal regeneration, Neural progenitor, Myelin, Mbp

## Abstract

**Introduction:**

Stem cell therapy using neural progenitor cells (NPCs) shows promise in mitigating the debilitating effects of spinal cord injury (SCI). Notably, myelin stimulates axonal regeneration from mammalian NPCs. This led us to hypothesize that myelin-associated proteins may contribute to axonal regeneration from NPCs.

**Methods:**

We conducted an R-based bioinformatics analysis to identify key gene(s) that may participate in myelin-associated axonal regeneration from murine NPCs, which identified the serine protease myelin basic protein (Mbp). We employed E12 murine NPCs, E14 rat NPCs, and human iPSC-derived Day 1 NPCs (D1 hNPCs) with or without CRISPR/Cas9-mediated *Mbp* knockout in combination with rescue L1-70 overexpression, constitutively-active VP16-PPARγ2, or the PPARγ agonist ciglitazone. A murine dorsal column crush model of SCI utilizing porous collagen-based scaffolding (PCS)-seeded murine NPCs with or without stable *Mbp* overexpression was used to assess locomotive recovery and axonal regeneration in vivo.

**Results:**

Myelin promotes axonal outgrowth from NPCs in an Mbp-dependent manner and that Mbp’s stimulatory effects on NPC neurite outgrowth are mediated by Mbp’s production of L1-70. Furthermore, we determined that Mbp/L1-70’s stimulatory effects on NPC neurite outgrowth are mediated by PPARγ-based repression of neuron differentiation-associated gene expression and PPARγ-based Erk1/2 activation. In vivo, PCS-seeded murine NPCs stably overexpressing *Mbp* significantly enhanced locomotive recovery and axonal regeneration in post-SCI mice.

**Conclusions:**

We discovered that Mbp supports axonal regeneration from mammalian NPCs through the novel Mbp/L1cam/Pparγ signaling pathway. This study suggests that bioengineered, NPC-based interventions can promote axonal regeneration and functional recovery post-SCI.

**Supplementary Information:**

The online version contains supplementary material available at 10.1186/s13578-021-00584-7.

## Highlights

- Bioinformatics identified Mbp as a key gene in myelin-associated NPC axonal regeneration
- Myelin promotes axonal outgrowth from NPCs in an Mbp-dependent manner
- Mbp’s stimulatory effects on NPC neurite outgrowth are mediated by Mbp’s production of L1-70.
- Mbp/L1-70’s stimulatory effects on NPC neurite outgrowth are mediated by PPARγ.
- *Mbp*-overexpressing NPCs enhanced locomotive recovery and axonal regeneration in post-SCI mice.

## Background

Spinal cord injury (SCI) is characterized by the presence of a pathophysiological cascade induced through physical disruption of the spinal column [1]. Although SCI accounts for only 0.01% injury occurrence globally (700,000 diagnoses every year), SCI are physically debilitating and SCI per-patient care costs over $500,000 in the first year and around $100,000 annually for as long as the patient lives [2]. These factors result in large socioeconomic costs on healthcare systems globally.

Stem cell therapy has shown promise in helping to mitigate the effects of SCI by aiding surviving cell populations and regenerating neuronal and glial populations [3]. Thanks to recent progress in stem cell biology, it is now possible to produce terminally-differentiated homogeneous cell populations from totipotent or multipotent stem cell progenitors in vivo [3]. Neural progenitor cells (NPCs) grafted into lesions on rodent spinal cords result in the formation of new axons of up to 50 mm in length, a distance approximately ten thousand times greater than normal adult axons [4–7].

NPCs can form axons prior to myelination during central nervous system (CNS) embryonic development. Myelin-associated proteins—such as ephrins, myelin-associated glycoprotein (MAG), Nogo, netrin, and oligodendrocyte myelin glycoprotein (OMgp)—inhibit axonal regeneration from adult neurons [8]. Experimental interventions targeting these myelin-associated molecules have had success in increasing the growth of adult axons, resulting in hundreds of new axons in spinal cord white matter [8]. In contrast, myelin has been shown to stimulate axonal regeneration from mammalian NPCs [8]. This evidence that myelin can stimulate axonal regrowth from NPCs led us to hypothesize that myelin-associated proteins may contribute to axonal regeneration from NPCs.

In this study, we conducted an in silico bioinformatics analysis to identify key gene(s) that may participate in myelin-associated axonal regeneration from murine NPCs, which identified the serine protease myelin basic protein (Mbp). We also present in vitro and in vivo evidence that supports axonal regeneration from mammalian NPCs through the novel Mbp/L1 cell adhesion molecule (L1cam)/Pparγ signaling pathway.

## Results

### Bioinformatics analysis identifies Mbp as a key myelin‐induced transcript in NPCs

**Fig. 1:**
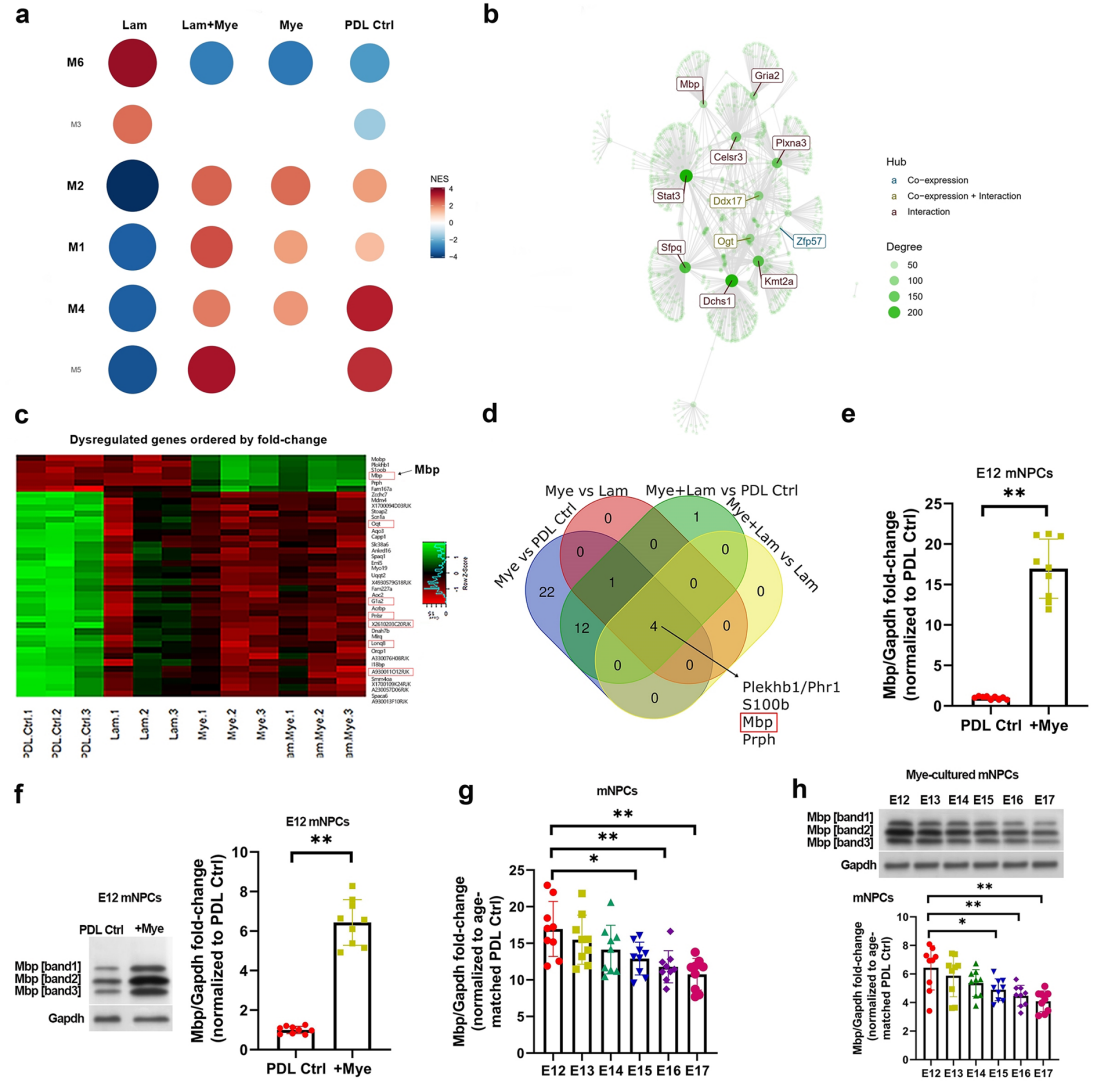
R-based bioinformatics analysis of E12 mNPCs subjected to different substrates. R-based bioinformatics analysis of published RNA-seq data (GEO acc no. GSE98974) from spinal cord-derived E12 mouse neural progenitor cells (E12 mNPCs) that were cultured on Poly-d-lysine control (PDL Ctrl), laminin (Lam), myelin (Mye), or Mye+Lam substrate [false discovery rate (FDR) < 0.1, n = 3 replicates/condition]. **a** Gene set enrichment analysis (GSEA)-based identification of six discreet gene co-expression modules (M1-6). Red coloring denotes a positive NES score, while blue coloring denotes a negative NES score. **b** The network plot and associated hub genes for the M6 gene co-expression module. **c** Heatmap of differentially-expressed genes (DEGs) ordered by descending log2 fold-change. Upregulation is denoted by green coloring, while downregulation is denoted by red coloring. **e** Venn diagram visualizing overlapping upregulated DEGs associated with Mye substrate. **f** qPCR of Mbp mRNA expression and **g** immunoblotting of Mbp in whole-cell lysates from E12 mNPCs on PDL Ctrl or myelin (Mye) substrates. Gapdh is the housekeeping and loading control. **h** qPCR of Mbp mRNA expression and **i** immunoblotting of Mbp protein expression in mNPCs reveals maturation-based Mbp downregulation on Mye substrate. All panels report means ± standard deviations (SDs). *n* = 3 embryos/genotype × 3 wells/embryo. *P < 0.05, **P < 0.01 [**e**, **f** Student’s t-test; **g**, **h** one-way ANOVA, post-hoc Tukey’s test]

We conducted an R-based bioinformatics analysis on previously published RNA-seq data (GEO accession no. GSE98974 [8]) derived from murine E12 spinal cord-derived NPCs (E12 mNPCs) that had been plated for 48 h on laminin (Lam), myelin (Mye), myelin+laminin (Mye +Lam), and PDL control (PDL Ctrl) substrate. These E12 mNPCs show similar neurite outgrowth profiles and responses to myelin stimulation as rat E14 spinal cord-derived NPCs (E14 rNPCs) and human iPSC-derived Day 1 NPCs (D1 hNPCs). We employed the R-based CemiTool package to ascertain the gene co-expression modules and pathways that differentiate the whole transcriptomes induced by the four substrates. We detected six distinct gene modules (Module [M]1–6) annotated by the mouse Reactome database (Fig. 1a). Based on the normalized enrichment scores (NES), four modules—M1, M2, M4, and M6  were enriched in the Mye-treated groups as compared to the non-Mye groups (Fig. 1a). Notably, the network diagrams of the four modules identified Mbp as a M6 hub gene (Fig. 1b, Additional file 1: Fig. S1A–D). GSEA revealed that M1 was enriched for various neurotransmitter release pathways, M2 was enriched for various protein folding and transport pathways, M4 was enriched for various transcriptional and translational pathways, and M6 was enriched for various FGFR signaling pathways (Additional file 1: Fig. S2A–D). DEG analysis revealed 6 upregulated and 33 downregulated genes in response to Mye substrate (Fig. 1d).

In order to specifically identify myelin-induced transcripts, we conducted a Venn analysis of upregulated DEGs from four comparisons: Mye vs. PDL Ctrl, Mye vs. Lam, Mye+Lam vs. PDL Ctrl, and Mye+Lam vs. Lam (Fig. 1e). We found four myelin-induced transcripts that were common across all comparisons: Plekhb1, S100b, Mbp, and Prph. Of these four transcripts, only Mbp was a member of an aforementioned gene module (i.e., M6). Therefore, we selected Mbp as a key myelin-induced transcript in NPCs for further investigation.

In order to validate our in silico findings, we employed qPCR and immunoblotting to measure myelin-induced Mbp mRNA and protein upregulation, respectively, in E12 mNPCs after a 48-h in vitro culture on myelin substrate (Fig. 1f, g). Notably, immunoblotting using a pan-Mbp antibody recognized three major bands (14, 18, and 21 kDa) for the Mbp protein (Fig. 1g). Moreover, qPCR and immunoblotting were performed to dynamically track Mbp mRNA and protein expression in maturing mNPCs (i.e., E12 to E17). The myelin-mediated increases in Mbp mRNA and protein levels gradually reduced on mNPC maturation from E12 to E17 (Fig. 1h, i).

### Myelin stimulates axonal outgrowth from NPCs in a mbp‐dependent manner

Three mammalian NPC models—E12 mNPCs, E14 rNPCs, or human iPSC-derived D1 hNPCs were utilized to better understand the mechanism(s) behind any Mbp-mediated effects on myelin-induced NPC dendrite regeneration. The selection of these particular NPC models was based on previous research on NPC axonal regeneration [8]. These NPCs, with or without CRISPR/Cas9-mediated *Mbp* knockout, were plated on Mye or PDL Ctrl substrates for 48 h. Western blotting validation of *Mbp* knockout using a pan-Mbp antibody recognized three major bands (14, 18, and 21 kDa) in WT NPCs, while no bands were detectable in *Mbp* knockout cells (Additional file 1: Fig. S3A–C). Neurite extensions were analyzed using anti-βIII-tubulin antibody staining.

**Fig. 2:**
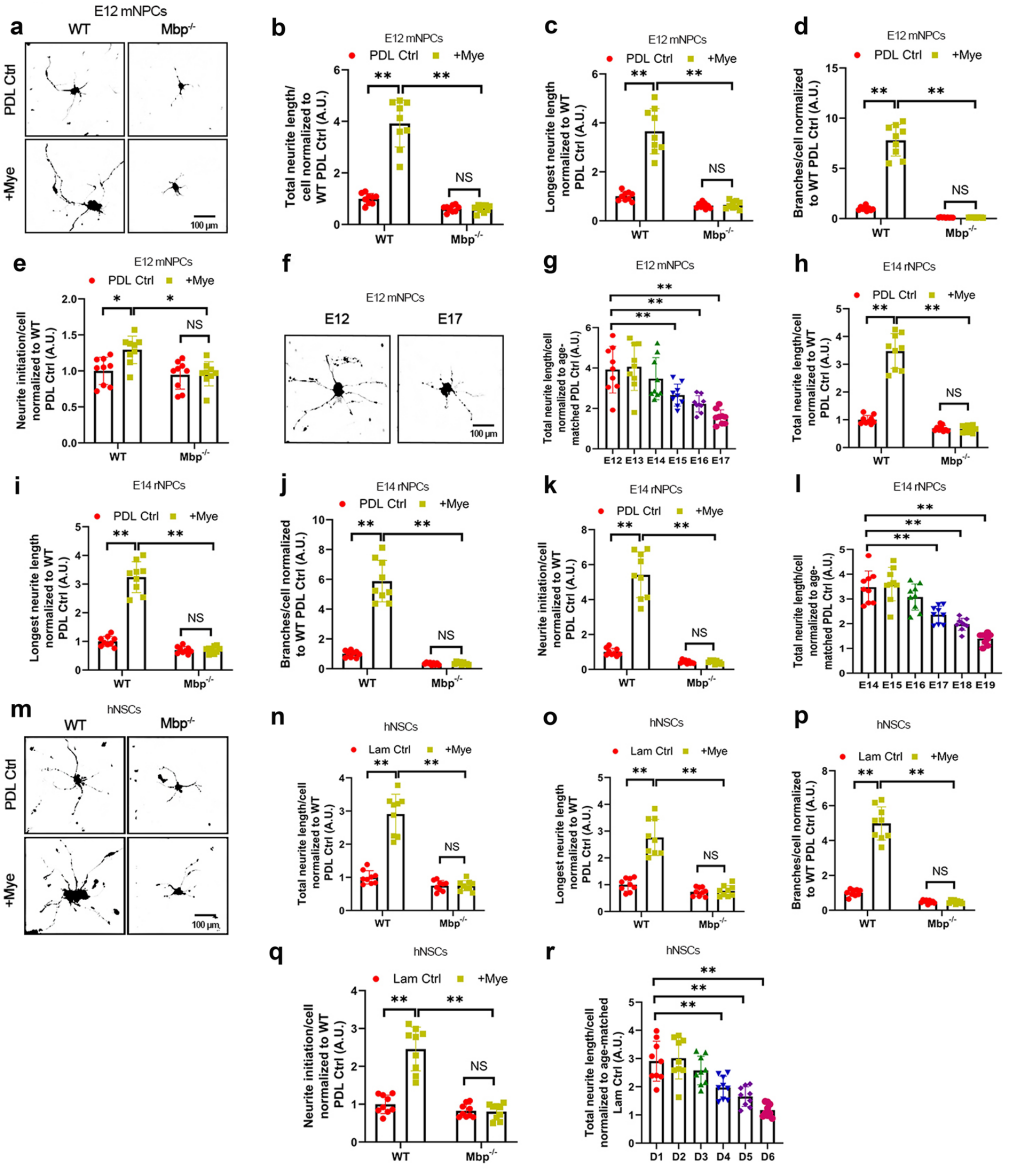
Mbp necessary for myelin-induced dendrite regeneration in NPCs.** a–g** Analysis of neuritogenesis in wild-type (WT) or Mbp-knockout (*Mbp*^*−/−*^) spinal cord-derived E12 mouse neural progenitor cells (E12 mNPCs) after 48 h of Poly-d-lysine control (PDL Ctrl) or mouse myelin (Mye) substrate culture. WT PDL Ctrl was used to normalize values for all experiments. **a** Representative images of axonal βIII-tubulin labeling in E12 mNPCs. Scale bar: 30 μm. **b** Total length of neurites per cell, **c** longest neurite per cell, **d** neurite branching for each cell, and **e** neurite initiation per cell. **f**, **g** Total length of neurites per cell from E12 to E17. Scale bars, 30 μm (**f**). **h–l** Analysis of neuritogenesis in WT or *Mbp*^*−/−*^ spinal cord-derived E14 rat neural progenitor cells (E14 rNPCs) after 48 h of PDL Ctrl or rat Mye substrate culture. WT PDL Ctrl was used to normalize values for all experiments. **h** Total length of neurites per cell, **i** longest neurite per cell, **j** neurite branching for each cell, and **k** neurite initiation per cell. **l** Total length of neurites per cell from E14 to E19. **m–r** Analysis of neuritogenesis in D1 WT or *Mbp*^*−/−*^ NPCs derived from human iPSCs (D1 hNPCs) after 48 h of Lam Ctrl or rat Mye substrate culture. As Lam substrate is required to culture hNPCs, Lam Ctrl was used to normalize values for each experiment. **m** Representative images of axonal βIII-tubulin labeling in E12 mNPCs. Scale bar: 20 μm. **n** Total length of neurites per cell, **o** longest neurite per cell, **p** neurite branching for each cell, and **q** neurite initiation per cell. **r** Total length of neurites per cell from D1 to D6. All panels report means ± standard deviations (SDs). *n* = 3 embryos/genotype × 3 wells/embryo. *P < 0.05, **P < 0.01 [two-way ANOVA, post-hoc Tukey’s test]

mNPCs displayed enhanced neurite lengths in the presence of Mye substrate (Fig. 2a–c). Mye substrate also stimulated greater neurite branching and initiation in mNPCs (Fig. 2d, e). Moreover, neurite outgrowth from mNPCs were dynamically measured from E12 to E17; Mye-induced outgrowth peaked at E12-13 and steadily declined thereafter (Fig. 2f, g). This finding was in agreement with the age-associated decline in Mbp expression (Fig. 1h, i). We found a similar pattern of findings in E14 rNPCs and D1 hNPCs cultured on rat Mye substrate (Fig. 2h–r). Notably, Mye-induced enhancements in neurite outgrowth and branching were abrogated by Mbp knockout in all three NPC models.

Mature neurons display myelin-based inhibition of neurite outgrowth in vitro [9]. If Mbp is essential to myelin-induced neurite outgrowth, Mbp overexpression may rescue myelin-based inhibition of neurite outgrowth in mature NPCs. Transfected E12 mNPCs were cultured on PDL Ctrl substrate for six days in order to induce maturation. The mature mNPCs were then re-plated on a myelin substrate. Mature mNPCs displayed myelin-based inhibition of neurite outgrowth, while Mbp overexpression partially rescued myelin-based inhibition (Additional file 1: Fig. S4A–E). We found a similar pattern of findings in E14 rNPCs and D1 hNPCs (Additional file 1: Fig. S4F–O). This data indicates that myelin promotes axonal outgrowth from NPCs in an Mbp-dependent manner.

### Mbp stimulates axonal outgrowth from NPCs via cleaving extracellular L1cam to produce L1-70

Mbp has been shown to be released into the extracellular space of adult mammalian neurons [10]. Mbp possesses serine protease activity and cleaves the extracellular protein L1 cell adhesion molecule (L1cam) to produce the 70-kDa L1cam fragment (L1-70), which potentiates L1cam-dependent neurite outgrowth in adult mammalian neurons [10]. Therefore, we hypothesized that Mbp’s serine protease activity would enhance extracellular L1cam cleavage and downstream neurite outgrowth in NPCs.

**Fig. 3:**
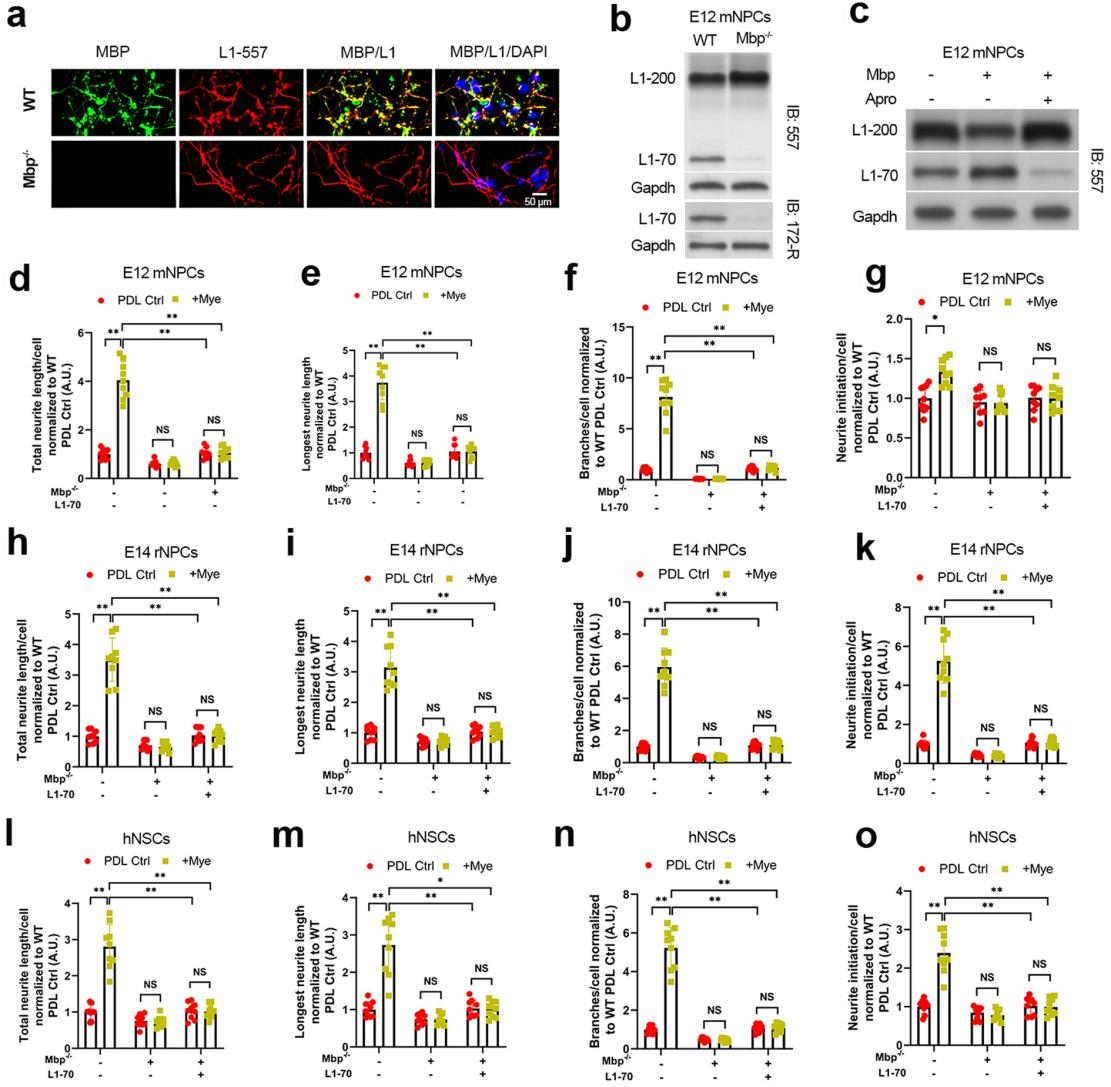
Mbp drives myelin-induced NPC dendrite regeneration via cleaving extracellular L1cam to produce L1-70. **a** Wild-type (WT) and Mbp-null (*Mbp*^*−/−*^) spinal cord-derived E12 mouse neural progenitor cells (E12 mNPCs) were stained with L1cam antibody-557 followed by pan-MBP antibody staining, fixation, Cy2-and Cy3-labeled secondary antibody staining, and DAPI (blue). Scale bars: 10 μm. **b** Immunoblotting of whole-cell lysates from WT and *Mbp*^*−/−*^ E12 mNPCs with either L1cam antibody-557 or antibody-172-R. Gapdh used as a loading control. **c** Whole-cell lysates from WT E12 mNPCs transfected with Mbp and treated with aprotinin were subjected to L1cam antibody-557 immunoblotting. Gapdh used as a loading control. **d–o** Analysis of neuritogenesis in **d–g** WT or *Mbp*^*−/−*^ E12 mNPCs, **h**–**k** WT or *Mbp*^*−/−*^ spinal cord-derived E14 rat neural progenitor cells (E14 rNPCs), and **l–o** D1 WT or *Mbp*^*−/−*^ NPCs derived from human iPSCs (D1 hNPCs) transduced with pcDNA3-Ctrl or pcDNA3-L1-70 after 48 h of Poly-d-lysine control (PDL Ctrl) or myelin (Mye) substrate culture. WT PDL Ctrl was used to normalize values for mNPCs and rNPCs. WT Lam Ctrl was used to normalize values for hNPCs. All panels report means ± standard deviations (SDs). *n* = 3 embryos/genotype × 3 wells/embryo. *P < 0.05, **P < 0.01 [two-way ANOVA, post-hoc Tukey’s test]

We first analyzed the localization of Mbp and L1cam in mNPCs by fluorescent immunostaining. Employing the pan-Mbp antibody and L1cam antibody-557 against mNPCs cultured on myelin substrate, we observed pronounced Mbp staining localized on the cell surface of WT mNPCs (Fig. 3a). We also observed co-localization of Mbp and L1cam at the cell surface of WT mNPCs. However, Mbp staining was absent in *Mbp* knockout mNPCs. These results indicate that Mbp and L1cam are extracellularly localized in NPCs.

Next, we investigated Mbp’s proteolytic processing of L1cam in E12 mNPCs with or without CRISPR/Cas9-mediated *Mbp* knockout cultured on the myelin substrate. Immunoblotting analysis using the L1cam antibody-557 revealed downregulated L1-70 and upregulated parental L1-200 by *Mbp* knockout (Fig. 3b). Also, the L1cam antibody 172-R directed against L1cam’s intracellular domain confirmed that L1-70 was downregulated by *Mbp* knockout (Fig. 3b). We then assessed whether *Mbp* overexpression enhances L1-70 production in E12 mNPCs. *Mbp* overexpression enhanced L1-70 production and reduced parental L1-200 expression, while the serine protease inhibitor aprotinin abolished these effects (Fig. 3c). These results show that Mbp’s serine protease activity promotes L1-70 production in NPCs.

To assess if L1-70 functions as a downstream intermediary for Mbp’s neuritogenic effects, neurite outgrowth was analyzed in E12 mNPCs, E14 rNPCs, or D1 hNPCs with or without CRISPR/Cas9-mediated *Mbp* knockout combined with L1-70 rescue overexpression. The reduction in neurite outgrowth by *Mbp* knockout was rescued by addition of L1-70 overexpression in E12 mNPCs (Fig. 3d–g), E14 rNPCs (Fig. 3h–k), and D1 hNPCs (Fig. 3l–o). These results indicate that Mbp’s stimulatory effects on neurite outgrowth in NPCs are mediated by Mbp’s production of L1-70 and establish the presence of a neuritogenic Mbp/L1-70 axis in NPCs.

### Mbp/L1-70 axis stimulates axonal outgrowth from NPCs via PPARγ

L1-70, which possesses the nuclear receptor co-activator motif LXXLL (L_1136_LILL), has been shown to translocate to the nucleus and bind to the nuclear receptor PPARγ, thereby regulating PPARγ target gene transactivation in mouse neurons [11]. Moreover, the PPARγ agonists ciglitazone and pioglitazone promote neurite outgrowth in neuroblastoma cells [12, 13]. This led us to hypothesize that the Mbp/L1-70 axis promotes axonal outgrowth from NPCs via stimulating PPARγ activation.

**Fig. 4:**
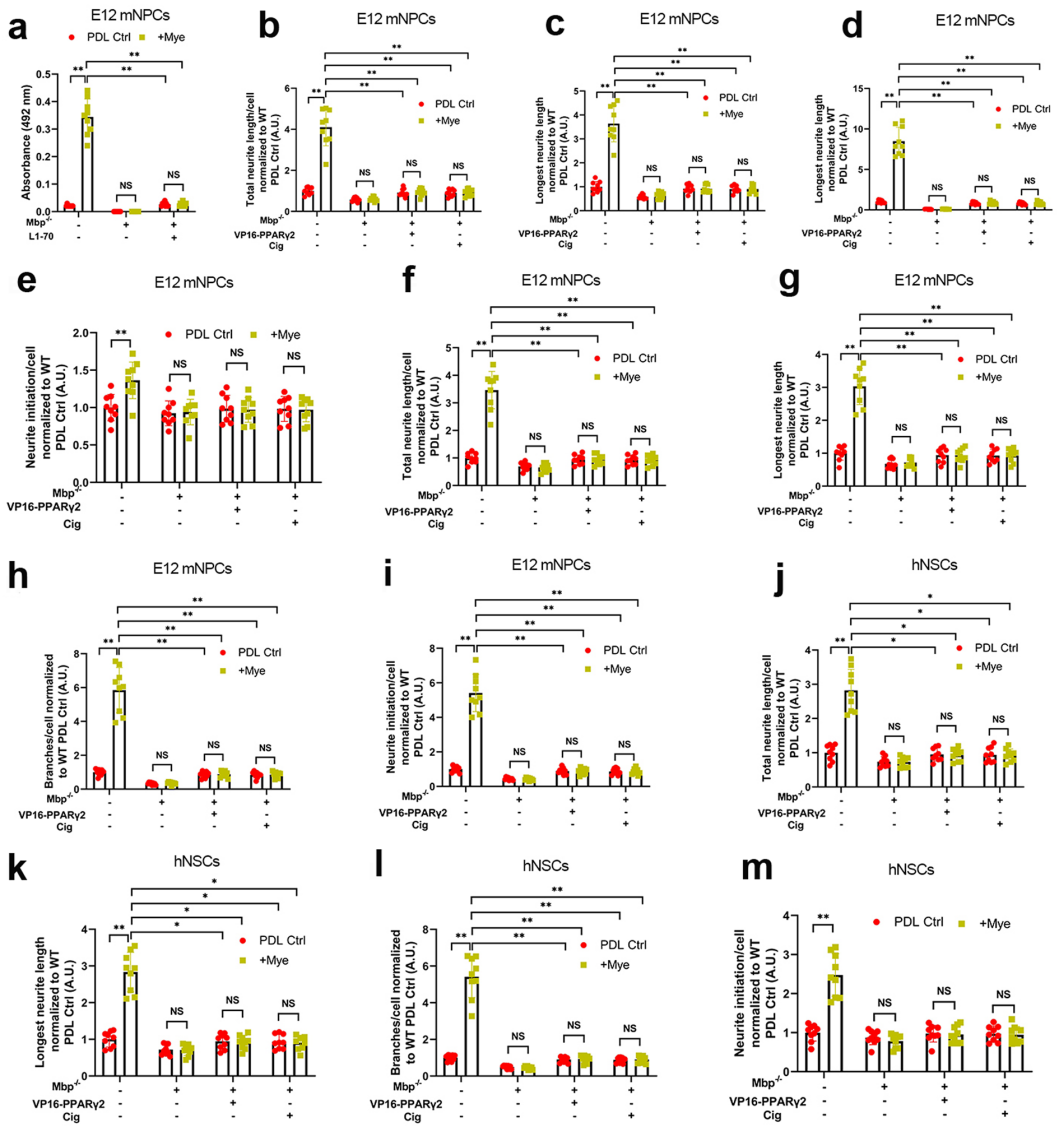
The Mbp/L1-70 axis drives myelin-induced NPC dendrite regeneration via PPARγ. **a** Nuclear fractions from wild-type (WT) and Mbp-null (*Mbp*^*−/−*^) spinal cord-derived E12 mouse neural progenitor cells (E12 mNPCs) transduced with pcDNA3-Ctrl or pcDNA3-L1-70 were applied to substrate-coated recombinant PPARγ for ELISA. Binding of nuclear L1-70 to recombinant PPARγ was assayed using L1cam antibody-172-R. **b–m** Analysis of neuritogenesis in **b–e** WT or *Mbp*^*−/−*^ E12 mNPCs, **f–i** WT or *Mbp*^*−/−*^ spinal cord-derived E14 rat neural progenitor cells (E14 rNPCs), and **j–m** D1 WT or *Mbp*^*−/−*^ NPCs derived from human iPSCs (D1 hNPCs) transduced with pcDNA3-Ctrl or pcDNA3-VP16-PPARγ2 and treated with the PPARγ agonist ciglitazone (1 µM) after 48 h of Poly-d-lysine control (PDL Ctrl) or myelin (Mye) substrate culture. WT PDL Ctrl was used to normalize values for mNPCs and rNPCs. WT Lam Ctrl was used to normalize values for hNPCs. All panels report means ± standard deviations (SDs). *n* = 3 embryos/genotype × 3 wells/embryo. *P < 0.05, **P < 0.01 [two-way ANOVA, post-hoc Tukey’s test]

We first validated whether nuclear L1-70 binds to PPARγ in E12 mNPCs with or without CRISPR/Cas9-mediated *Mbp* knockout cultured on the myelin substrate. Using ELISA, we applied the nuclear fraction from E12 mNPCs to substrate-coated recombinant PPARγ. Nuclear fractions from WT E12 mNPCs showed strong binding of nuclear L1-70 to recombinant PPARγ (Fig. 4a). However, the binding strength was attenuated by *Mbp* knockout and rescued by addition of L1-70 overexpression. These results indicate that Mbp’s generation of L1-70 drives nuclear L1-70’s binding to PPARγ in NPCs.

To assess if PPARγ functions as a downstream intermediary for Mbp/L1-70’s neuritogenic effects, neurite outgrowth was analyzed in E12 mNPCs, E14 rNPCs, or D1 hNPCs with or without CRISPR/Cas9-mediated *Mbp* knockout. The reduction in neurite outgrowth by *Mbp* knockout was rescued by constitutively-active VP16-PPARγ2 or ciglitazone in E12 mNPCs (Fig. 4b–e), E14 rNPCs (Fig. 4f–i), and D1 hNPCs (Fig. 4j–m). These results indicate that Mbp/L1-70’s stimulatory effects on neurite outgrowth in NPCs are mediated by PPARγ activity.

### Mbp/L1-70 axis regulates expression of PPARγ’s neuron differentiation‐associated gene targets in NPCs

**Fig. 5:**
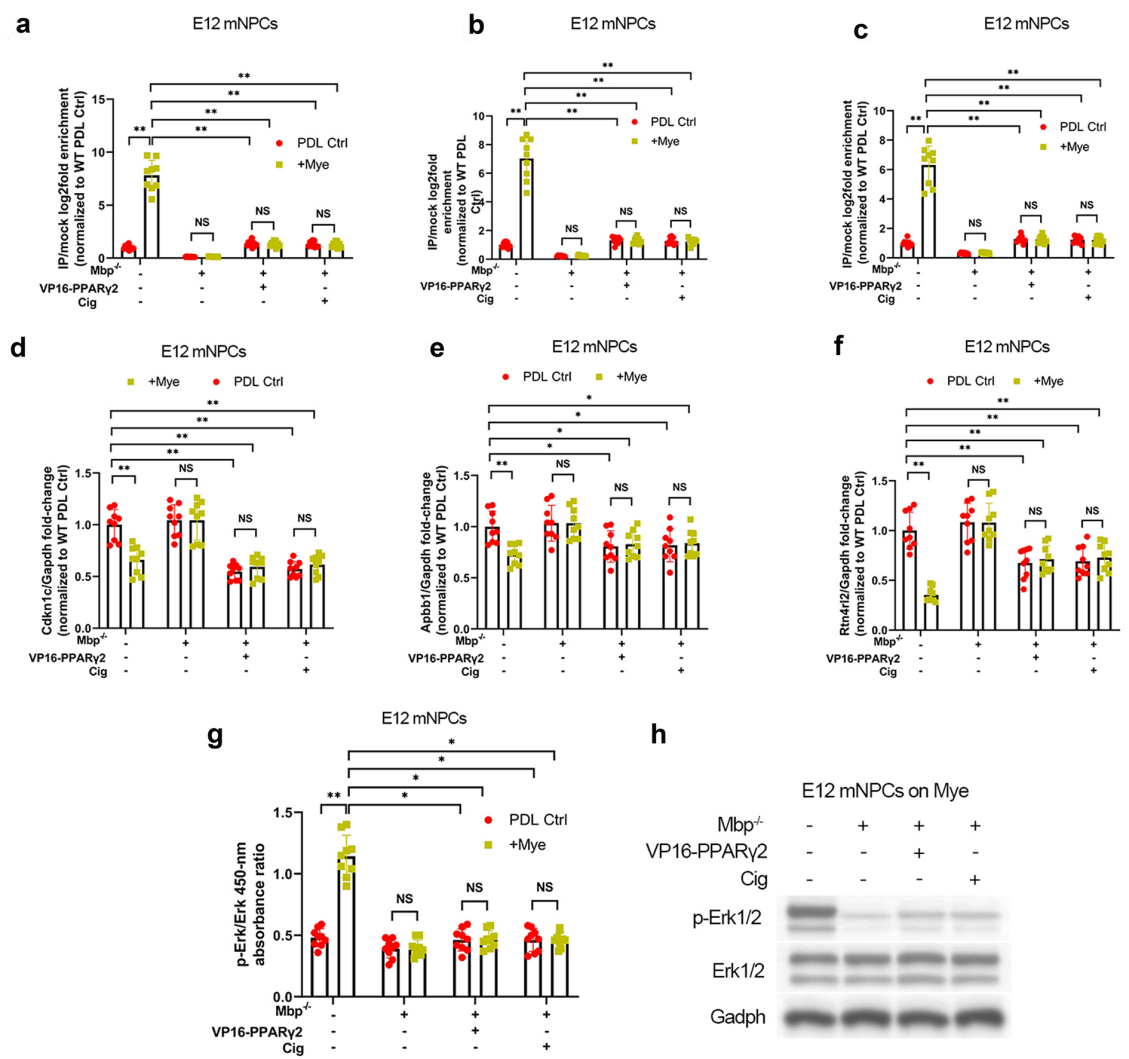
Mbp/L1-70 axis regulates expression of PPARγ’s dendrite regeneration-associated gene targets in NPCs. Wild-type (WT) and Mbp-null (*Mbp*^*−/−*^) spinal cord-derived E12 mouse neural progenitor cells (E12 mNPCs) transduced with pcDNA3-Ctrl or pcDNA3-VP16-PPARγ2 and treated with the PPARγ agonist ciglitazone (1 µM) after 48 h of Poly-d-lysine control (PDL Ctrl) or myelin (Mye) substrate culture. WT PDL Ctrl was used to normalize values. **a–c** PCR quantification of anti-PPARγ immunoprecipitated chromatin using site-specific primers. **d–f** qPCR of PPARγ target gene expression. **g**, **h** Activation of extracellular signal–regulated kinase 1/2 (Erk1/2) signaling assayed by **g** ELISA and **h** immunoblotting. All panels report means ± standard deviations (SDs). *n* = 3 embryos/genotype × 3 wells/embryo. *P < 0.05, **P < 0.01 [two-way ANOVA, post-hoc Tukey’s test]

Activated PPARγ typically functions as a transcriptional repressor by binding to target gene promoters and reducing their transactivation [12]. Notably, three PPARγ target genes are associated with neuron differentiation and are dysregulated in a rat dorsal root crush model, namely *Apbb1*, *Cdkn1c*, and *Rtn4rl2* [12]. To assess if PPARγ functions as a downstream repressor of these three target genes in NPCs, quantitative ChIP of Apbb1, Cdkn1c, and Rtn4rl2 was performed in E12 mNPCs with or without CRISPR/Cas9-mediated *Mbp* knockout. E12 mNPCs were stimulated with DMSO vehicle or ciglitazone; after 24 h, chromatin complexes were cross-linked and subjected to anti-PPARγ ChIP. We found specific binding interactions between PPARγ and the *Apbb1*, *Cdkn1c*, and *Rtn4rl2* gene promoters, which were downregulated by *Mbp* knockout and rescued by VP16-PPARγ2 or ciglitazone (Fig. 5a–c). qPCR analysis revealed that *Mbp* knockout significantly enhanced Apbb1, Cdkn1c, and Rtn4rl2 mRNA expression, which was rescued by VP16-PPARγ2 or ciglitazone (Fig. 5d–f). These results demonstrate that the Mbp/L1-70 axis inhibits PPARγ-mediated repression of neuron differentiation-associated gene expression.

PPARγ activity stimulates Erk1/2 (p42/p44 MAPK) activation [13], which has been shown to enhance neurite outgrowth in NPCs [8]. This led us to hypothesize that the Mbp/L1-70/PPARγ axis may upregulate Erk1/2 activation in NPCs. Therefore, we investigated Erk1/2 activation in E12 mNPCs with or without CRISPR/Cas9-mediated *Mbp* knockout. ELISA and immunoblotting at 6 h of substrate culture revealed that Erk1/2 phosphorylation was downregulated by *Mbp* knockout, which was rescued by VP16-PPARγ2 or ciglitazone (Fig. 5g, h). These results indicate that the Mbp/L1-70/PPARγ axis promotes neuritogenic Erk1/2 activation in NPCs.

### Stable *Mbp* overexpression in scaffolded mNPCs improves locomotive recovery and axonal regeneration post-SCI

mNPCs seeded on porous collagen-based scaffolding (PCS) in a murine dorsal column crush model of SCI (characterized by irreversible hind limb instability and coordination dysfunction) have been shown to significantly improve axonal regeneration and locomotive recovery in vivo [9]. Based on Mbp’s regenerative effects in vitro, we hypothesized that PCS-seeded mNPCs stably overexpressing *Mbp* would significantly enhance locomotive recovery in this murine model of SCI.

**Fig. 6:**
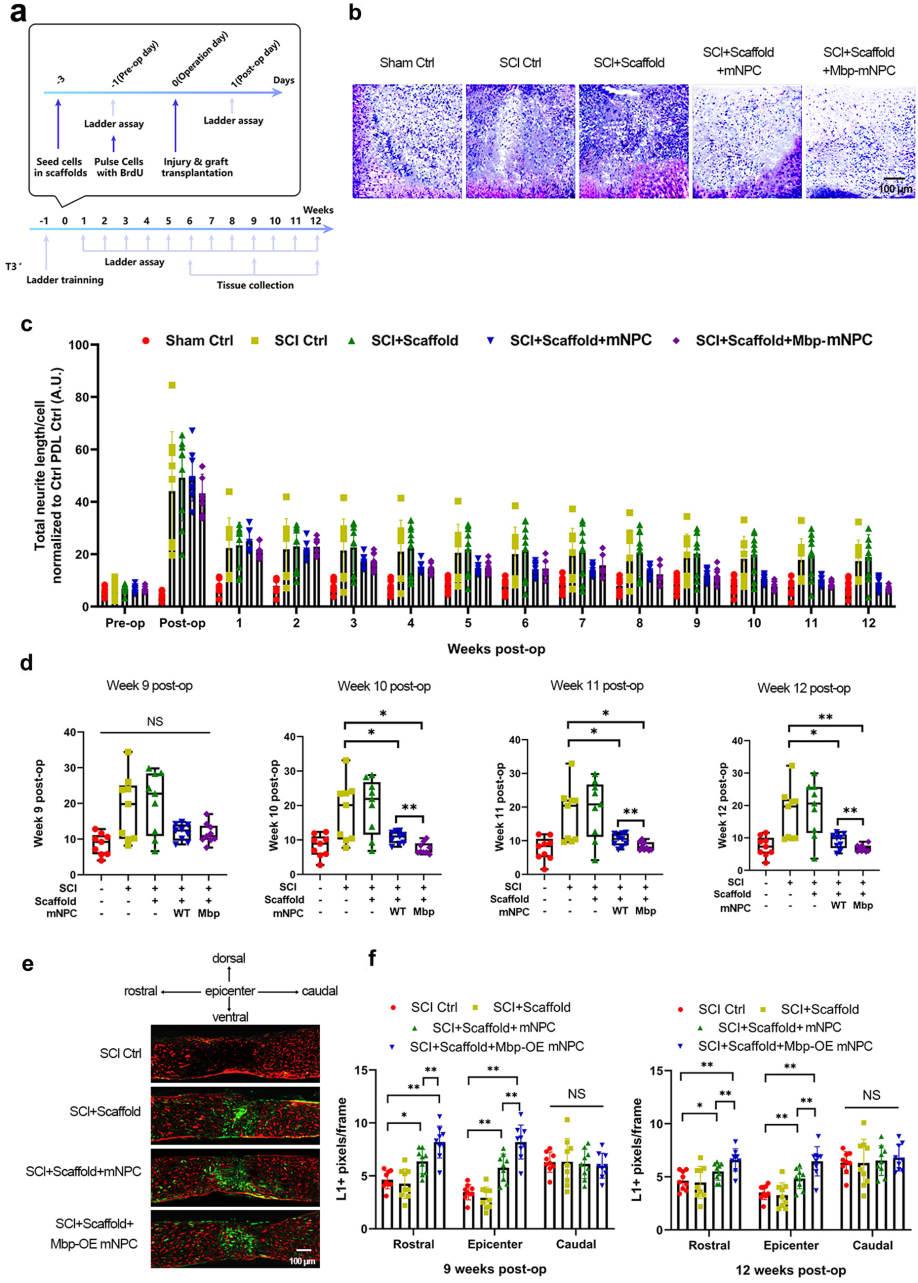
Stable *Mbp* overexpression in scaffolded mNPCs produces superior locomotive recovery and axonal regeneration in a murine model of SCI.** a** Experimental schematic overview of murine model of SCI detailing timepoints for spinal cord crush injury (SCI), grafting, locomotive fault analysis, and sacrifice for histological analysis. **b** H&E-stained parasagittal spinal cord sections 6 weeks post-SCI. Scale bar, 200 μm. **c** Quantification of fault rates by horizontal ladder walking assay post-SCI. **d** Boxplots of fault rates at 9, 10, 11, and 12 weeks post-SCI. **e** Representative immunofluorescent images of parasagittal spinal cord sections 6 weeks post-SCI stained for bovine collagen I, neurofilament heavy chain (NF), and L1-70. Scale bar, 200 μm. **f** Quantification of L1-70 + pixel density 9 weeks and 12 weeks post-SCI caudally, epicentrally, and rostrally to the SCI lesion. Results are normalized to the Sham Ctrl group. Panels report either means ± standard deviations (SDs) [bar and line charts] or medians ± interquartile ranges (IQRs) [boxplots]. *n* = 9 animals per cohort. *P < 0.05, **P < 0.01 [one-way ANOVA, post-hoc Tukey’s test]

The in vivo experiments consisted of five cohorts of mice (i.e., Sham Ctrl, SCI Ctrl, SCI+Scaffold, SCI+Scaffold+mNPC, and SCI+Scaffold+Mbp-mNPC), with eight mice per cohort (Fig. 6a). Post-mortem H&E staining of SCI lesion sections at 6 weeks, 9 weeks, and 12 weeks post-SCI revealed that mNPC-seeded and Mbp-mNPC-seeded scaffolds fully integrated into the surrounding nerve tissue (Fig. 6b). However, scaffold-only grafts had migrated from the SCI lesion site in 100% of mice at 6 weeks post-SCI injury (Fig. 6b).

Locomotive recovery following SCI was analyzed via the locomotive fault rate on the horizontal ladder walking assay over the 12-week period post-SCI. The fault rate remained unchanged in the Sham Ctrl group over the 12-week evaluation period (Fig. 6c). However, over the 12-week evaluation period, we detected a significant effect of SCI (*P* < 0.0001 [repeated measures two-way ANOVA], F[4,480] = 70.10). Starting at three weeks post-SCI, the locomotive performance in both mNPC groups improved over the other SCI groups (*P* < 0.0001 [repeated measures two-way ANOVA], F[3,384] = 27.26). Notably, the SCI+Scaffold+Mbp-mNPC group showed a significant superiority over the SCI+Scaffold+mNPC group at ten weeks, eleven weeks, and twelve weeks post-SCI (Fig. 6d).

Axon regeneration through the SCI lesion is necessary for locomotive recovery [9]. Therefore, we analyzed axonal regeneration following SCI via NF and L1-70 immunostaining at nine and 12 weeks post-SCI. Nine weeks post-SCI, immunostaining of the SCI Ctrl sections revealed weak L1-70 staining at the SCI lesion site (Fig. 6e) In contrast, the mNPC groups revealed significant numbers of L1-70 + axons at the lesion epicenter as well as rostrally and caudally of the SCI lesion site (Fig. 6e). At nine and 12 weeks post-SCI, both mNPC groups showed significantly greater L1-70 pixel density relative to the SCI Ctrl group rostrally and epicentrally, with the SCI+Scaffold+Mbp-mNPC group showing significant superiority over the SCI+Scaffold+mNPC group (Fig. 6f). These results reveal that PCS-seeded mNPCs stably overexpressing *Mbp* significantly enhance locomotive recovery and axonal regeneration in this murine model of SCI.

## Discussion

NPC-based regenerative therapies for SCI are aimed at stimulating NPC axons to grow into the host spinal cord tissue past the site of injury, thereby forming functional synaptic connections with the host neurons. Myelin—a multi-lamellar plasma membrane extension formed from oligodendrocytes that spirally wrap around the axon is a biologically-active substrate that supports axonal growth [14]. Groundbreaking work by Poplawski et al. has revealed that myelin augments neurite outgrowth from NPCs [8]. Here, we conducted an in silico bioinformatics analysis to identify key gene(s) that may participate in myelin-associated axonal regeneration from murine NPCs, which identified the serine protease Mbp. We also present in vitro and in vivo evidence that supports axonal regeneration from mammalian NPCs through the novel Mbp/L1cam/Pparγ signaling pathway.

In this study, *Mbp* was identified through bioinformatics analysis of published RNA-seq data (GEO accession no. GSE98974 [8]); follow-up validation through *Mbp-*knockout/*Mbp*-overexpression studies revealed that *Mbp* mediates myelin-induced neurite outgrowth from NPCs in vitro and axonal regeneration in a murine model of SCI. Mbp—a positively-charged, disordered polypeptide is a key structural component of myelin that resides between the negatively-charged, cytoplasmic leaflets of the myelin membrane [14]. Mbp’s interaction with these cytoplasmic leaflets triggers Mbp’s polymeric self-assembly, thereby expelling cytoplasm from the myelin sheath’s interior and promoting healthy membrane compaction [14]. In addition to this structural function, the 21.5-kDa isoform of Mbp associates with the C-terminus of dynamin I to create a catalytically-active serine protease [15]. On the cell surface, this catalytically-active Mbp isoform cleaves the parental L1cam glycoprotein L1-200 at the arginine 687 residue (within the first FNIII domain of L1cam) to generate L1-70 (a transmembrane C-terminal fragment) and L1-135 [15] (an extracellular N-terminal fragment). Here, we found that Mbp’s stimulatory effects on neurite outgrowth in NPCs are mediated by Mbp’s production of L1-70. Indeed, several lines of published research support that Mbp-generated L1-70 promotes neuritogenesis and axonal regeneration. In vitro, Mbp-generated L1-70 promotes neurite outgrowth in cerebellar neurons, which is abrogated by Mbp knockdown, blockade with an anti-Mbp antibody, or delivery of a L1cam peptide with a competitive Mbp cleavage site [10]. In mice, Mbp knockout or targeted mutagenesis of Mbp’s proteolytically-active site eliminates L1-70 generation [10], while applying Mbp onto injured spinal cord tissue or viral-mediated Mbp overexpression in injured spinal cord tissue promotes L1-70 expression, re-myelination, and functional recovery post-SCI, while blockade with an anti-Mbp antibody or viral-mediated overexpression of proteolytically-inactive Mbp produces the opposite effects [16]. Moreover, the small-molecule L1cam mimic phenelzine upregulates spinal cord L1cam, improves hind limb functioning, and stimulates axonal regeneration at four and 5 weeks post-SCI in mice [17]. Furthermore, viral-mediated L1cam overexpression in a spinal cord transection (SCT) rat model reveals that post-SCI generation of diffusible L1cam fragments improves neurite outgrowth and myelination [18].

Delving further into Mbp/L1-70’s mechanism of action, we found that Mbp/L1-70’s stimulatory effects on neurite outgrowth in NPCs are mediated by PPARγ activity. Previous research has shown that L1-70 is imported into the nucleus, wherein its nuclear receptor co-activator motif LXXLL (L_1136_LILL) mediates its binding to various nuclear receptors, including PPARγ, estrogen receptor (ER) α and β, and retinoid X receptor β (RXRβ) [11]. In utero mutations in this LXXLL motif within murine embryos impairs cerebellar synaptic connectivity, motor coordination, and cognition in adulthood [11], suggesting that L1-70’s interaction with these nuclear receptors regulates neural plasticity pathways. Indeed, we found that the Mbp/L1-70 axis inhibits PPARγ-mediated repression of neuron differentiation-associated gene expression and promotes neuritogenic Erk1/2 activation in NPCs.

There are several limitations to this study. First, although *Mbp* knockout produced profound neurite growth reductions in NPCs cultured on myelin, it is reasonable to assume that there may be other myelin-associated proteins that significantly impact neurite outgrowth. Second, we focused on Mbp/L1-70’s stimulatory effects on PPARγ activity as a key regulator of neurite outgrowth from NPCs. It is possible that other downstream target(s) of the Mbp/L1-70 axis may have an impact on neurite outgrowth from NPCs. As a more complete profile of the molecular players involved in myelin-mediated NPC axonal regeneration would greatly aid therapeutic development for SCI, these issues require further evaluation.

In conclusion, we report that Mbp supports axonal regeneration from mammalian NPCs through the novel Mbp/L1cam/Pparγ signaling pathway. This study also indicates that bioengineered, NPC-based interventions can promote axonal regeneration and functional recovery post-SCI.

## Methods

The detailed procedures regarding the R-based bioinformatics analysis, mice and rat subjects, myelin extraction, preparation and maintenance of cell culture plates, generation and culture of NPCs, plasmid construction and transfection, maturation assays, fluorescent immunostaining, quantitative polymerase chain reaction (qPCR), enzyme-linked immunosorbent assay (ELISA), immunoblotting, and the murine dorsal column crush model of SCI are provided in the Supplementary Methods. Unless stated otherwise, data are reported as means ± standard deviations (SDs). A two-tailed, unpaired Student’s *t-*test, one-way analysis of variance (ANOVA) with Tukey’s post-hoc test, or two-way ANOVA with Tukey’s post-hoc test (Additional file 1: Table S1) were employed for statistical comparisons between experimental groups as indicated. A repeated measures two-way ANOVA was used to analyze changes in locomotive recovery. All statistical analyses utilized a strict significance criterion (*P < 0.05, **P < 0.01).

## Supplementary Information

**Additional file 1.** Supplementary Figures S1–S4.

## Data Availability

The data used to support the findings of this study are available from the corresponding author upon request.

## Abbreviations

CNS: Central nervous system
DEGs: Differentially-expressed genes
ER: Estrogen receptor
Erk1/2: Extracellular signal-regulated kinase
FDR: False discovery rate
IHC: Immunohistochemical
L1cam: L1 cell adhesion molecule
Lam: Laminin
Mye: Myelin
Mbp: Myelin basic protein
MAG: Myelin-associated glycoprotein
NPCs: Neural progenitor cells
NF: Neurofilament
NES: Normalized enrichment scores
OMgp: Oligodendrocyte myelin glycoprotein
PBS: phosphate-bufferedsaline
PCS: porous collagen-based scaffolding
qPCR: Quantitative polymerase chain reaction
SCI: Spinal cord injury
WT: Wild-type
