# Supplementary material for: Myelin basic protein enhances axonal regeneration from neural progenitor cells
Source: Cell Biosci. 2021 Apr 29;11:80. doi: 10.1186/s13578-021-00584-7 (PMC8082623; doi:10.1186/s13578-021-00584-7)
Supplement: Supplementary file 1 — Additional file 1. Supplementary Figures S1–S4. [file 13578_2021_584_MOESM1_ESM.docx]

**SUPPLEMENTARY INFORMATION FOR**

**Myelin basic protein enhances axonal regeneration from neural progenitor cells**

**SUPPLEMENTARY METHODS**

**Bioinformatics analysis**

R was used to conduct all bioinformatics analyses. For modular analysis of the previously-published RNA-seq data (GEO accession no. GSE98974 ) ([1](#_ENREF_1)), the CemiTools package in R was used with default parameters and the mouse Reactome database for annotation. Following median-based filtering and normalization, the Limma package in R was used to determine the differentially-expressed genes (DEGs) using a false discovery rate (FDR) of < 10% (*q* < 0.1) and adjusted *p*-value < 0.05.

**Animals**

Wild-type (WT) Fischer 344 rats and C57BL/6 mice as well as Shiverer (*Mbp^shi/shi^*) mice on a C57BL/6 background ([2](#_ENREF_2)) were obtained from the Jackson Laboratory. Animals were maintained on a 12 h light/dark cycle and had *ad libitum* access to standard chow and tap water. Enrollment into experimental groups was randomized by a random number generator, and researchers were blinded to experimental conditions during data processing and analysis.

*Mbp^-/-^* Fischer 344 rats were constructed by Charles River (www.criver.com) using a conventional CRISPR/Cas9 procedure ([3](#_ENREF_3)). Briefly, Cas9 mRNA and sgRNAs were cytoplasmically co-injected into Fischer 344 rat zygotes using standard microinjection methods. The mosaic founders were then analyzed for off-target edits using a previously described algorithm ([3](#_ENREF_3)). The mosaic founders lacking off-target edits were then bred to WT Fischer 344 rat counterparts in order to generate F1 heterozygous progeny for intercrossing. For embryonic tissue harvesting, gravid female rats and mice were humanely sacrificed and subjected to embryo extraction as previously described ([4](#_ENREF_4)).

**Myelin extraction**

Sucrose-gradient centrifugation was used to extract rat myelin or mouse myelin from frozen spinal cords of WT Fischer 344 rats or WT C57BL/6 mice, respectively ([5](#_ENREF_5)). To assess myelin protein concentrations, a Bradford protein assay (Bio-Rad) was used.

**Preparation and maintenance of cell culture plates**

For mouse and rat NPC culture plates, a pre-coating of poly-D-lysine (PDL, 20 µg/ml in phosphate-buffered saline (PBS), Sigma-Aldrich) was applied overnight to six-well tissue culture plates at room temperature. Some wells also received a pre-coating of mouse myelin or rat myelin (10 µg/ml in PBS) overnight at 4°C. All plates were washed with media three times prior to culturing.

For human NPC culture plates, an overnight pre-coating of six-well tissue culture plates at 37°C was performed using 40 µg/ml poly-ornithine in sterile water (Sigma-Aldrich). The next morning, the plates were washed three times using sterile water and then coated with either 10 µg/ml rat myelin in PBS (myelin-treated) or an equivalent volume of PBS vehicle (control) and left overnight at 4°C. The two sets of plates were then washed with PBS three times and treated with a coating of 5 µg/ml laminin in media at room temperature for 4 hours to create laminin control plates or myelin-treated plates. All plates were washed with media three times prior to culturing.

**Generation and culture of NPCs**

Spinal cord-derived NPCs were prepared from E14-E19 Fischer 344 rats (rNPCs) or E12-E17 C57BL/6 mice (mNPCs) as previously described ([6](#_ENREF_6)) . Human iPSCs were purchased from Thermo Fisher. We employed a plasmid electroporation-mediated CRISPR/Cas9 method ([7](#_ENREF_7)) for knockout of *MBP* (NM_001025090.2) in human iPSCs using the following sgRNA (GPP sgRNA Designer, MIT Broad Institute): 5′-GTCCGGCCGTGTGACTTCTG-3′. Human iPSC-derived Day 1 NPCs (D1 hNPCs) were produced as described previously ([8](#_ENREF_8)).

NPCs were plated at 10,000 cells/250 µl Neurobasal+B27 (standard media) in 48-well plates. For substrate plating experiments, NPCs were plated in substrate-coated six-well tissue culture plates (3 × 10^6^ cells per well) and cultured in 5% CO_2_ at 37°C for 48 hours unless otherwise indicated. Two hours after plating, mouse myelin or rat myelin (10 µg/ml in media) was added to the media. For selected experiments, the PPARγ agonist ciglitazone (1 μM in DMSO) or an equivalent volume of DMSO vehicle (all Sigma-Aldrich) was added to the culture medium. The mitosis assay was performed by adding 10 µM BrdU to the culture medium.

**Plasmid construction and transfection**

Full-length cDNAs of mouse/rat/human *Mbp* (NM_001025251.2/NM_001025291.1/ NM_001025081.2), the L1-70 segment of mouse/rat/human *L1cam* (NM_008478.3 /NM_017345.1/NM_001278116.2), and the constitutively-active PPARγ construct *VP16-PPARγ2* ([9](#_ENREF_9)) in addition to their PCR amplification primers were purchased from Sino Biological (Beijing, China). The PCR amplification products were separated by agarose gel electrophoresis, purified, and ligated into a pcDNA3 plasmid according to the manufacturer's instructions (Invitrogen).

E12 mNPCs, E14 rNPCs, or D1 hNPCs were transfected with a 4D-Nucleofector system and the P3 Primary Cell Kit using a cell volume of 3 million per cuvette (Lonza). Briefly, 3 µg of either empty pcDNA3 backbone (pcDNA3-Ctrl), pcDNA3-Mbp, pcDNA3-L1-70, or pcDNA3-VP16-PPARγ2 was electroporated into the NPCs using a 16-well Nucleocuvette Strip. The transfected NPCs were plated on substrate-coated six-well plates as described above.

**Maturation assay**

The *in vitro* maturation assay was performed using E12 mNPCs, E14 NPCs, or D1 hNPCs plated in PDL-coated six-well plates and allowed to culture under standard conditions for six days. After the culture period, the cells were treated with a PBS wash (calcium- and magnesium-free) three times and trypsin-digested in a 0.25% trypsin solution at 37°C for 10 minutes. The cells were then dissociated in standard media with added 10% fetal bovine serum and plated on the indicated substrates.

**Fluorescent immunostaining**

As described previously ([10](#_ENREF_10)), cells were fixed and stained with anti-βIII-tubulin (1:2000, Promega). Cells were imaged at either 4- or 10-times magnification using ImageXpress. Neurite outgrowth was quantified automatically using Molecular Devices’ MetaXpress software.

**Quantitative polymerase chain reaction (qPCR)**

The RNeasy Mini kit (Qiagen) was used to isolate total RNA from NPC cultures. PrimeScript RT Master Mix (Perfect Real Time, Clontech) was used to perform the reverse transcription reactions. qSTAR qPCR primers were purchased from Origene; the primer sequences are fully detailed on Origene’s website (www.origene.com/products/gene-expression/qpcr/primer-pairs). qPCR reactions were conducted with SensiMix SYBR Master Mix (Origene) on a LightCycler 480 with LightCycler 480 v1.5 software (Roche). Glyceraldehyde-3-phosphate dehydrogenase (Gapdh) was used as a housekeeping control.

**Enzyme-linked immunosorbent assay (ELISA) and immunoblotting**

ELISA for Erk1/2 phosphorylation was carried out using the procedure outlined by the Erk1/2 Instant One ELISA Kit (eBioscience). Immunoblots were performed according to established protocols ([11](#_ENREF_11)) and stained with the following primary antibodies: anti-Gapdh housekeeping control (1:10000, Sigma-Aldrich), anti-Mbp (1:1000, ab7349, Abcam), anti-L1cam (antibody 557) and anti-L1-70 (antibody 172-R) (both 1 μg/ml, Zentrum für Molekulare Neurobiologie, Universitätsklinikum Hamburg-Eppendorf, Hamburg, Germany), anti-phospho-Erk1/2 (1:1000, #9101, CST), and anti-Erk1/2 (1:1000, #4695, CST). SuperSignal West Dura ECL substrate (Thermo Fisher Scientific) was used to develop blots, they were then imaged using the ChemiDoc MP imaging system (Bio-Rad). Blot densitometry data was quantified using Image Lab software (Bio-Rad).

**Murine crush model of SCI**

mNPCs stably overexpressing *Mbp* were generated by lentiviral infection of the gene expression vector pCDH-MSCV-MCS-EF1-Puro (System Biosciences) as previously described ([12](#_ENREF_12)). Porous collagen sheets were fabricated as described previously ([13](#_ENREF_13)); a simple biopsy punch was used to construct the 3 mm-diameter cylindrical collagen scaffolds.

The SCI murine model was constructed as previously described ([13](#_ENREF_13)). Briefly, male C57BL/6 mice (6-8 weeks old) were acclimated to our lab conditions and walked through the ladder platform for a one-week period. Three days before surgery, 3 × 10^4^ mNPCs were seeded onto 1.0 mm × 1.0 mm × 1.5 mm cylindrical collagen scaffolds using standard media. The dorsal column crush procedure was performed by fine forceps placed into the spinal cord white matter rostral to the T13 segment as previously described ([13](#_ENREF_13)). Grafts were washed in PBS for 30 min prior to implantation. In the SCI group, no graft was applied to the SCI lesion. In the SCI+Scaffold group, a cell-free scaffold was placed onto the SCI lesion. In the SCI+Scaffold+mNPC and SCI+Scaffold+Mbp-mNPC groups, the indicated mNPC-seeded collagen scaffolds were placed onto the SCI lesion.

For analysis of locomotive recovery, mice were video-recorded while walking along a horizontal ladder as previously described ([13](#_ENREF_13)); blinded investigators then analyzed the video footage for locomotive faults (i.e., hind limb misplaced upon, slipping off, or missing the rod). Only serially-consecutive steps were included in the fault analysis (i.e., the last step before and the first step after stopping were excluded). Locomotive faults were classified on a seven-point scale: complete miss (0), deep slip (1), slight slip (2), replacement (3), correction (4), partial placement (5), or correct placement (6). The fault rate was defined as the percentage of steps classified as faults (i.e., 0-5). All SCI subjects displayed a fault rate > 15% one day post-SCI. Each subject was run on this ladder assay five times in each session; results from each run were then averaged to achieve the subject’s fault rate. After 6 weeks, 9 weeks, or 12 weeks following the SCI procedure, subjects were anesthetized with 2.5:1 isoflurane:oxygen mix and transcardially perfused with ice-cold heparinized saline (10 U/ml) then 4% PFA in PBS. H&E and immunohistochemical (IHC) analysis on 20-μm parasagittal sections prepared from the 2-mm diameter portion surrounding the SCI lesion was performed as previously described ([13](#_ENREF_13)). The following primary antibodies for IHC analysis were diluted in PBST and applied overnight at 4°C: anti-L1-70 (antibody 172-R) (1 μg/ml); anti-neurofilament heavy chain (NF) (1:1000; ab4680, Abcam); and anti-bovine collagen I (1:100; ab34710, Abcam). Fluorophore-conjugated secondary antibodies (1:1000, Thermo) were applied for one hour at room temperature. Following two PBS washes, the stained slides were mounted, DAPI counterstained, and visualized by inverted confocal microscopy. For L1cam pixel analysis, the immunostained sections were imaged caudally, epicentrally, and rostrally to the SCI lesion as previously described ([13](#_ENREF_13)). Following adjustments for background signal intensity and channel intensity, the average percentages of L1cam-positive pixels in the SCI lesion epicenter and immediately caudal and rostral to the SCI lesion boundary were calculated from twelve randomly-selected fields per section (i.e., four fields for each direction) × six sections per subject.

**Statistical analysis**

Unless stated otherwise, data are reported as means ± standard deviations (SDs). A two-tailed, unpaired Student’s *t-*test or one-way analysis of variance (ANOVA) with Tukey’s post-hoc test for experiments involving two groups or more than two groups, respectively. A repeated measures two-way ANOVA was used to analyze changes in locomotive recovery. All statistical analyses utilized a strict significance criterion (**P<*0.05, ***P*<0.01).

**SUPPLEMENTARY TABLES**

**Table S1. Descriptive statistics from two-way analysis of variance (ANOVA) testing†**

| **Figure panel** | **Two-way ANOVA results** | | | | | |
| --- | --- | --- | --- | --- | --- | --- |
| **2B** |  |  |  |  |  |  |
| *Source of Variation* | *SS* | *df* | *MS* | *F* | *P-value* | *F crit* |
| Substrate | 19.18 | 1.00 | 19.18 | 85.03 | <0.01** | 4.15 |
| Genotype | 30.70 | 1.00 | 30.70 | 136.10 | <0.01** | 4.15 |
| Interaction | 19.26 | 1.00 | 19.26 | 85.38 | <0.01** | 4.15 |
| Within | 7.22 | 32.00 | 0.23 |  |  |  |
| **Total** | **76.36** | **35.00** |  |  |  |  |
|  |  |  |  |  |  |  |
| **2C** |  |  |  |  |  |  |
| *Source of Variation* | *SS* | *df* | *MS* | *F* | *P-value* | *F crit* |
| Substrate | 16.23 | 1.00 | 16.23 | 70.03 | <0.01** | 4.15 |
| Genotype | 25.85 | 1.00 | 25.85 | 111.55 | <0.01** | 4.15 |
| Interaction | 15.57 | 1.00 | 15.57 | 67.16 | <0.01** | 4.15 |
| Within | 7.42 | 32.00 | 0.23 |  |  |  |
| **Total** | **65.06** | **35.00** |  |  |  |  |
|  |  |  |  |  |  |  |
| **2D** |  |  |  |  |  |  |
| *Source of Variation* | *SS* | *df* | *MS* | *F* | *P-value* | *F crit* |
| Substrate | 103.95 | 1.00 | 103.95 | 166.02 | <0.01** | 4.15 |
| Genotype | 166.31 | 1.00 | 166.31 | 265.64 | <0.01** | 4.15 |
| Interaction | 103.98 | 1.00 | 103.98 | 166.07 | <0.01** | 4.15 |
| Within | 20.04 | 32.00 | 0.63 |  |  |  |
| **Total** | **394.28** | **35.00** |  |  |  |  |
|  |  |  |  |  |  |  |
| **2E** |  |  |  |  |  |  |
| *Source of Variation* | *SS* | *df* | *MS* | *F* | *P-value* | *F crit* |
| Substrate | 0.22 | 1.00 | 0.22 | 6.18 | 0.02* | 4.15 |
| Genotype | 0.34 | 1.00 | 0.34 | 9.71 | <0.01** | 4.15 |
| Interaction | 0.18 | 1.00 | 0.18 | 5.15 | 0.03* | 4.15 |
| Within | 1.11 | 32.00 | 0.03 |  |  |  |
| **Total** | **1.85** | **35.00** |  |  |  |  |
|  |  |  |  |  |  |  |
|  |  |  |  |  |  |  |
| **2H** |  |  |  |  |  |  |
| *Source of Variation* | *SS* | *df* | *MS* | *F* | *P-value* | *F crit* |
| Substrate | 13.60 | 1.00 | 13.60 | 122.33 | <0.01** | 4.15 |
| Genotype | 21.84 | 1.00 | 21.84 | 196.38 | <0.01** | 4.15 |
| Interaction | 14.04 | 1.00 | 14.04 | 126.24 | <0.01** | 4.15 |
| Within | 3.56 | 32.00 | 0.11 |  |  |  |
| **Total** | **53.04** | **35.00** |  |  |  |  |
|  |  |  |  |  |  |  |
| **2I** |  |  |  |  |  |  |
| *Source of Variation* | *SS* | *df* | *MS* | *F* | *P-value* | *F crit* |
| Substrate | 11.38 | 1.00 | 11.38 | 130.43 | <0.01** | 4.15 |
| Genotype | 18.19 | 1.00 | 18.19 | 208.44 | <0.01** | 4.15 |
| Interaction | 11.26 | 1.00 | 11.26 | 129.00 | <0.01** | 4.15 |
| Within | 2.79 | 32.00 | 0.09 |  |  |  |
| **Total** | **43.63** | **35.00** |  |  |  |  |
|  |  |  |  |  |  |  |
| **2J** |  |  |  |  |  |  |
| *Source of Variation* | *SS* | *df* | *MS* | *F* | *P-value* | *F crit* |
| Substrate | 53.89 | 1.00 | 53.89 | 107.78 | <0.01** | 4.15 |
| Genotype | 86.10 | 1.00 | 86.10 | 172.18 | <0.01** | 4.15 |
| Interaction | 53.12 | 1.00 | 53.12 | 106.23 | <0.01** | 4.15 |
| Within | 16.00 | 32.00 | 0.50 |  |  |  |
| **Total** | **209.11** | **35.00** |  |  |  |  |
|  |  |  |  |  |  |  |
| **2K** |  |  |  |  |  |  |
| *Source of Variation* | *SS* | *df* | *MS* | *F* | *P-value* | *F crit* |
| Substrate | 43.79 | 1.00 | 43.79 | 98.47 | <0.01** | 4.15 |
| Genotype | 70.11 | 1.00 | 70.11 | 157.69 | <0.01** | 4.15 |
| Interaction | 44.14 | 1.00 | 44.14 | 99.26 | <0.01** | 4.15 |
| Within | 14.23 | 32.00 | 0.44 |  |  |  |
| **Total** | **172.27** | **35.00** |  |  |  |  |
|  |  |  |  |  |  |  |

| **2N** |  |  |  |  |  |  |
| --- | --- | --- | --- | --- | --- | --- |
| *Source of Variation* | *SS* | *df* | *MS* | *F* | *P-value* | *F crit* |
| Substrate | 8.18 | 1.00 | 8.18 | 73.13 | <0.01** | 4.15 |
| Genotype | 13.10 | 1.00 | 13.10 | 117.05 | <0.01** | 4.15 |
| Interaction | 8.22 | 1.00 | 8.22 | 73.46 | <0.01** | 4.15 |
| Within | 3.58 | 32.00 | 0.11 |  |  |  |
| **Total** | **33.08** | **35.00** |  |  |  |  |
|  |  |  |  |  |  |  |
| **2O** |  |  |  |  |  |  |
| *Source of Variation* | *SS* | *df* | *MS* | *F* | *P-value* | *F crit* |
| Substrate | 7.29 | 1.00 | 7.29 | 50.35 | <0.01** | 4.15 |
| Genotype | 11.57 | 1.00 | 11.57 | 79.93 | <0.01** | 4.15 |
| Interaction | 6.76 | 1.00 | 6.76 | 46.66 | <0.01** | 4.15 |
| Within | 4.63 | 32.00 | 0.14 |  |  |  |
| **Total** | **30.25** | **35.00** |  |  |  |  |
|  |  |  |  |  |  |  |
| **2P** |  |  |  |  |  |  |
| *Source of Variation* | *SS* | *df* | *MS* | *F* | *P-value* | *F crit* |
| Substrate | 35.35 | 1.00 | 35.35 | 147.40 | <0.01** | 4.15 |
| Genotype | 56.66 | 1.00 | 56.66 | 236.28 | <0.01** | 4.15 |
| Interaction | 35.98 | 1.00 | 35.98 | 150.05 | <0.01** | 4.15 |
| Within | 7.67 | 32.00 | 0.24 |  |  |  |
| **Total** | **135.66** | **35.00** |  |  |  |  |
|  |  |  |  |  |  |  |
| **2Q** |  |  |  |  |  |  |
| *Source of Variation* | *SS* | *df* | *MS* | *F* | *P-value* | *F crit* |
| Substrate | 4.68 | 1.00 | 4.68 | 40.24 | <0.01** | 4.15 |
| Genotype | 7.53 | 1.00 | 7.53 | 64.73 | <0.01** | 4.15 |
| Interaction | 4.92 | 1.00 | 4.92 | 42.35 | <0.01** | 4.15 |
| Within | 3.72 | 32.00 | 0.12 |  |  |  |
| **Total** | **20.85** | **35.00** |  |  |  |  |
|  |  |  |  |  |  |  |

| **S4B** |  |  |  |  |  |  |
| --- | --- | --- | --- | --- | --- | --- |
| *Source of Variation* | *SS* | *df* | *MS* | *F* | *P-value* | *F crit* |
| Substrate | 13.85 | 1.00 | 13.85 | 220.56 | <0.01** | 4.15 |
| Genotype | 7.24 | 1.00 | 7.24 | 115.34 | <0.01** | 4.15 |
| Interaction | 2.31 | 1.00 | 2.31 | 36.71 | <0.01** | 4.15 |
| Within | 2.01 | 32.00 | 0.06 |  |  |  |
| **Total** | **25.41** | **35.00** |  |  |  |  |
|  |  |  |  |  |  |  |
| **S4C** |  |  |  |  |  |  |
| *Source of Variation* | *SS* | *df* | *MS* | *F* | *P-value* | *F crit* |
| Substrate | 16.59 | 1.00 | 16.59 | 208.33 | <0.01** | 4.15 |
| Genotype | 11.48 | 1.00 | 11.48 | 144.20 | <0.01** | 4.15 |
| Interaction | 3.71 | 1.00 | 3.71 | 46.56 | <0.01** | 4.15 |
| Within | 2.55 | 32.00 | 0.08 |  |  |  |
| **Total** | **34.32** | **35.00** |  |  |  |  |
|  |  |  |  |  |  |  |
| **S4D** |  |  |  |  |  |  |
| *Source of Variation* | *SS* | *df* | *MS* | *F* | *P-value* | *F crit* |
| Substrate | 71.10 | 1.00 | 71.10 | 224.71 | <0.01** | 4.15 |
| Genotype | 60.03 | 1.00 | 60.03 | 189.72 | <0.01** | 4.15 |
| Interaction | 34.12 | 1.00 | 34.12 | 107.84 | <0.01** | 4.15 |
| Within | 10.13 | 32.00 | 0.32 |  |  |  |
| **Total** | **175.38** | **35.00** |  |  |  |  |
|  |  |  |  |  |  |  |
| **S4E** |  |  |  |  |  |  |
| *Source of Variation* | *SS* | *df* | *MS* | *F* | *P-value* | *F crit* |
| Substrate | 0.45 | 1.00 | 0.45 | 14.13 | <0.01** | 4.15 |
| Genotype | 0.35 | 1.00 | 0.35 | 10.96 | <0.01** | 4.15 |
| Interaction | 0.00 | 1.00 | 0.00 | 0.00 | 0.99 | 4.15 |
| Within | 1.01 | 32.00 | 0.03 |  |  |  |
| **Total** | **1.81** | **35.00** |  |  |  |  |
|  |  |  |  |  |  |  |

| **S4G** |  |  |  |  |  |  |
| --- | --- | --- | --- | --- | --- | --- |
| *Source of Variation* | *SS* | *df* | *MS* | *F* | *P-value* | *F crit* |
| Substrate | 15.14 | 1.00 | 15.14 | 202.75 | <0.01** | 4.15 |
| Genotype | 10.04 | 1.00 | 10.04 | 134.44 | <0.01** | 4.15 |
| Interaction | 3.19 | 1.00 | 3.19 | 42.73 | <0.01** | 4.15 |
| Within | 2.39 | 32.00 | 0.07 |  |  |  |
| **Total** | **30.76** | **35.00** |  |  |  |  |
|  |  |  |  |  |  |  |
| **S4H** |  |  |  |  |  |  |
| *Source of Variation* | *SS* | *df* | *MS* | *F* | *P-value* | *F crit* |
| Substrate | 12.38 | 1.00 | 12.38 | 159.46 | <0.01** | 4.15 |
| Genotype | 9.04 | 1.00 | 9.04 | 116.42 | <0.01** | 4.15 |
| Interaction | 2.18 | 1.00 | 2.18 | 28.11 | <0.01** | 4.15 |
| Within | 2.49 | 32.00 | 0.08 |  |  |  |
| **Total** | **26.09** | **35.00** |  |  |  |  |
|  |  |  |  |  |  |  |
| **S4I** |  |  |  |  |  |  |
| *Source of Variation* | *SS* | *df* | *MS* | *F* | *P-value* | *F crit* |
| Substrate | 41.66 | 1.00 | 41.66 | 190.13 | <0.01** | 4.15 |
| Genotype | 33.13 | 1.00 | 33.13 | 151.22 | <0.01** | 4.15 |
| Interaction | 15.94 | 1.00 | 15.94 | 72.76 | <0.01** | 4.15 |
| Within | 7.01 | 32.00 | 0.22 |  |  |  |
| **Total** | **97.75** | **35.00** |  |  |  |  |
|  |  |  |  |  |  |  |
| **S4J** |  |  |  |  |  |  |
| *Source of Variation* | *SS* | *df* | *MS* | *F* | *P-value* | *F crit* |
| Substrate | 36.23 | 1.00 | 36.23 | 157.20 | <0.01** | 4.15 |
| Genotype | 27.41 | 1.00 | 27.41 | 118.94 | <0.01** | 4.15 |
| Interaction | 12.97 | 1.00 | 12.97 | 56.28 | <0.01** | 4.15 |
| Within | 7.37 | 32.00 | 0.23 |  |  |  |
| **Total** | **83.98** | **35.00** |  |  |  |  |
|  |  |  |  |  |  |  |
| **S4L** |  |  |  |  |  |  |
| *Source of Variation* | *SS* | *df* | *MS* | *F* | *P-value* | *F crit* |
| Substrate | 9.93 | 1.00 | 9.93 | 128.49 | <0.01** | 4.15 |
| Genotype | 6.76 | 1.00 | 6.76 | 87.51 | <0.01** | 4.15 |
| Interaction | 1.48 | 1.00 | 1.48 | 19.14 | <0.01** | 4.15 |
| Within | 2.47 | 32.00 | 0.08 |  |  |  |
| **Total** | **20.65** | **35.00** |  |  |  |  |
|  |  |  |  |  |  |  |
| **S4M** |  |  |  |  |  |  |
| *Source of Variation* | *SS* | *df* | *MS* | *F* | *P-value* | *F crit* |
| Substrate | 8.70 | 1.00 | 8.70 | 120.86 | <0.01** | 4.15 |
| Genotype | 6.07 | 1.00 | 6.07 | 84.37 | <0.01** | 4.15 |
| Interaction | 1.14 | 1.00 | 1.14 | 15.83 | <0.01** | 4.15 |
| Within | 2.30 | 32.00 | 0.07 |  |  |  |
| **Total** | **18.21** | **35.00** |  |  |  |  |
|  |  |  |  |  |  |  |
| **S4N** |  |  |  |  |  |  |
| *Source of Variation* | *SS* | *df* | *MS* | *F* | *P-value* | *F crit* |
| Substrate | 30.40 | 1.00 | 30.40 | 213.07 | <0.01** | 4.15 |
| Genotype | 23.18 | 1.00 | 23.18 | 162.47 | <0.01** | 4.15 |
| Interaction | 9.89 | 1.00 | 9.89 | 69.34 | <0.01** | 4.15 |
| Within | 4.56 | 32.00 | 0.14 |  |  |  |
| **Total** | **68.03** | **35.00** |  |  |  |  |
|  |  |  |  |  |  |  |
| **S4O** |  |  |  |  |  |  |
| *Source of Variation* | *SS* | *df* | *MS* | *F* | *P-value* | *F crit* |
| Substrate | 6.75 | 1.00 | 6.75 | 188.32 | <0.01** | 4.15 |
| Genotype | 4.28 | 1.00 | 4.28 | 119.52 | <0.01** | 4.15 |
| Interaction | 0.72 | 1.00 | 0.72 | 20.18 | <0.01** | 4.15 |
| Within | 1.15 | 32.00 | 0.04 |  |  |  |
| **Total** | **12.90** | **35.00** |  |  |  |  |
|  |  |  |  |  |  |  |

| **3D** |  |  |  |  |  |  |
| --- | --- | --- | --- | --- | --- | --- |
| *Source of Variation* | *SS* | *df* | *MS* | *F* | *P-value* | *F crit* |
| Substrate | 14.15 | 1.00 | 14.15 | 111.96 | <0.01** | 4.04 |
| Genotype | 36.07 | 2.00 | 18.04 | 142.73 | <0.01** | 3.19 |
| Interaction | 27.53 | 2.00 | 13.76 | 108.91 | <0.01** | 3.19 |
| Within | 6.07 | 48.00 | 0.13 |  |  |  |
| **Total** | **83.81** | **53.00** |  |  |  |  |
|  |  |  |  |  |  |  |
| **3E** |  |  |  |  |  |  |
| *Source of Variation* | *SS* | *df* | *MS* | *F* | *P-value* | *F crit* |
| Substrate | 11.59 | 1.00 | 11.59 | 106.17 | <0.01** | 4.04 |
| Genotype | 30.01 | 2.00 | 15.00 | 137.45 | <0.01** | 3.19 |
| Interaction | 22.23 | 2.00 | 11.12 | 101.84 | <0.01** | 3.19 |
| Within | 5.24 | 48.00 | 0.11 |  |  |  |
| **Total** | **69.07** | **53.00** |  |  |  |  |
|  |  |  |  |  |  |  |
| **3F** |  |  |  |  |  |  |
| *Source of Variation* | *SS* | *df* | *MS* | *F* | *P-value* | *F crit* |
| Substrate | 77.67 | 1.00 | 77.67 | 156.97 | <0.01** | 4.04 |
| Genotype | 196.80 | 2.00 | 98.40 | 198.87 | <0.01** | 3.19 |
| Interaction | 151.39 | 2.00 | 75.70 | 152.99 | <0.01** | 3.19 |
| Within | 23.75 | 48.00 | 0.49 |  |  |  |
| **Total** | **449.60** | **53.00** |  |  |  |  |
|  |  |  |  |  |  |  |
| **3G** |  |  |  |  |  |  |
| *Source of Variation* | *SS* | *df* | *MS* | *F* | *P-value* | *F crit* |
| Substrate | 0.15 | 1.00 | 0.15 | 6.28 | 0.02* | 4.04 |
| Genotype | 0.48 | 2.00 | 0.24 | 10.28 | <0.01** | 3.19 |
| Interaction | 0.36 | 2.00 | 0.18 | 7.64 | <0.01** | 3.19 |
| Within | 1.12 | 48.00 | 0.02 |  |  |  |
| **Total** | **3.21** | **53.00** |  |  |  |  |
|  |  |  |  |  |  |  |

| **3H** |  |  |  |  |  |  |
| --- | --- | --- | --- | --- | --- | --- |
| *Source of Variation* | *SS* | *df* | *MS* | *F* | *P-value* | *F crit* |
| Substrate | 8.89 | 1.00 | 8.89 | 80.36 | <0.01** | 4.04 |
| Genotype | 24.76 | 2.00 | 12.38 | 111.88 | <0.01** | 3.19 |
| Interaction | 19.45 | 2.00 | 9.73 | 87.90 | <0.01** | 3.19 |
| Within | 5.31 | 48.00 | 0.11 |  |  |  |
| **Total** | **58.42** | **53.00** |  |  |  |  |
|  |  |  |  |  |  |  |
| **3I** |  |  |  |  |  |  |
| *Source of Variation* | *SS* | *df* | *MS* | *F* | *P-value* | *F crit* |
| Substrate | 7.02 | 1.00 | 7.02 | 70.12 | <0.01** | 4.04 |
| Genotype | 18.33 | 2.00 | 9.16 | 91.55 | <0.01** | 3.19 |
| Interaction | 13.73 | 2.00 | 6.87 | 68.60 | <0.01** | 3.19 |
| Within | 4.80 | 48.00 | 0.10 |  |  |  |
| **Total** | **43.89** | **53.00** |  |  |  |  |
|  |  |  |  |  |  |  |
| **3J** |  |  |  |  |  |  |
| *Source of Variation* | *SS* | *df* | *MS* | *F* | *P-value* | *F crit* |
| Substrate | 37.28 | 1.00 | 37.28 | 142.17 | <0.01** | 4.04 |
| Genotype | 96.32 | 2.00 | 48.16 | 183.67 | <0.01** | 3.19 |
| Interaction | 73.09 | 2.00 | 36.54 | 139.36 | <0.01** | 3.19 |
| Within | 12.59 | 48.00 | 0.26 |  |  |  |
| **Total** | **219.28** | **53.00** |  |  |  |  |
|  |  |  |  |  |  |  |
| **3K** |  |  |  |  |  |  |
| *Source of Variation* | *SS* | *df* | *MS* | *F* | *P-value* | *F crit* |
| Substrate | 26.45 | 1.00 | 26.45 | 109.70 | <0.01** | 4.04 |
| Genotype | 72.27 | 2.00 | 36.14 | 149.88 | <0.01** | 3.19 |
| Interaction | 55.13 | 2.00 | 27.57 | 114.33 | <0.01** | 3.19 |
| Within | 11.57 | 48.00 | 0.24 |  |  |  |
| **Total** | **165.43** | **53.00** |  |  |  |  |
|  |  |  |  |  |  |  |

| **3L** |  |  |  |  |  |  |
| --- | --- | --- | --- | --- | --- | --- |
| *Source of Variation* | *SS* | *df* | *MS* | *F* | *P-value* | *F crit* |
| Substrate | 4.69 | 1.00 | 4.69 | 51.18 | <0.01** | 4.04 |
| Genotype | 13.18 | 2.00 | 6.59 | 71.94 | <0.01** | 3.19 |
| Interaction | 10.03 | 2.00 | 5.02 | 54.77 | <0.01** | 3.19 |
| Within | 4.40 | 48.00 | 0.09 |  |  |  |
| **Total** | **32.30** | **53.00** |  |  |  |  |
|  |  |  |  |  |  |  |
| **3M** |  |  |  |  |  |  |
| *Source of Variation* | *SS* | *df* | *MS* | *F* | *P-value* | *F crit* |
| Substrate | 4.34 | 1.00 | 4.34 | 33.25 | <0.01** | 4.04 |
| Genotype | 12.37 | 2.00 | 6.19 | 47.40 | <0.01** | 3.19 |
| Interaction | 9.16 | 2.00 | 4.58 | 35.07 | <0.01** | 3.19 |
| Within | 6.27 | 48.00 | 0.13 |  |  |  |
| **Total** | **32.13** | **53.00** |  |  |  |  |
|  |  |  |  |  |  |  |
| **3N** |  |  |  |  |  |  |
| *Source of Variation* | *SS* | *df* | *MS* | *F* | *P-value* | *F crit* |
| Substrate | 26.70 | 1.00 | 26.70 | 124.42 | <0.01** | 4.04 |
| Genotype | 68.81 | 2.00 | 34.40 | 160.31 | <0.01** | 3.19 |
| Interaction | 53.54 | 2.00 | 26.77 | 124.73 | <0.01** | 3.19 |
| Within | 10.30 | 48.00 | 0.21 |  |  |  |
| **Total** | **159.34** | **53.00** |  |  |  |  |
|  |  |  |  |  |  |  |
| **3O** |  |  |  |  |  |  |
| *Source of Variation* | *SS* | *df* | *MS* | *F* | *P-value* | *F crit* |
| Substrate | 2.55 | 1.00 | 2.55 | 33.81 | <0.01** | 4.04 |
| Genotype | 7.79 | 2.00 | 3.90 | 51.71 | <0.01** | 3.19 |
| Interaction | 6.17 | 2.00 | 3.09 | 40.97 | <0.01** | 3.19 |
| Within | 3.62 | 48.00 | 0.08 |  |  |  |
| **Total** | **20.13** | **53.00** |  |  |  |  |
|  |  |  |  |  |  |  |

| **4A** |  |  |  |  |  |  |
| --- | --- | --- | --- | --- | --- | --- |
| *Source of Variation* | *SS* | *df* | *MS* | *F* | *P-value* | *F crit* |
| Substrate | 0.16 | 1.00 | 0.16 | 240.02 | <0.01** | 4.04 |
| Genotype | 0.35 | 2.00 | 0.17 | 259.15 | <0.01** | 3.19 |
| Interaction | 0.32 | 2.00 | 0.16 | 235.84 | <0.01** | 3.19 |
| Within | 0.03 | 48.00 | 0.00 |  |  |  |
| **Total** | **0.85** | **53.00** |  |  |  |  |
|  |  |  |  |  |  |  |
| **4B** |  |  |  |  |  |  |
| *Source of Variation* | *SS* | *df* | *MS* | *F* | *P-value* | *F crit* |
| Substrate | 11.27 | 1.00 | 11.27 | 90.40 | <0.01** | 3.99 |
| Genotype | 42.28 | 3.00 | 14.09 | 113.10 | <0.01** | 2.75 |
| Interaction | 32.11 | 3.00 | 10.70 | 85.89 | <0.01** | 2.75 |
| Within | 7.98 | 64.00 | 0.12 |  |  |  |
| **Total** | **93.63** | **71.00** |  |  |  |  |
|  |  |  |  |  |  |  |
| **4C** |  |  |  |  |  |  |
| *Source of Variation* | *SS* | *df* | *MS* | *F* | *P-value* | *F crit* |
| Substrate | 8.25 | 1.00 | 8.25 | 87.59 | <0.01** | 3.99 |
| Genotype | 31.53 | 3.00 | 10.51 | 111.63 | <0.01** | 2.75 |
| Interaction | 23.10 | 3.00 | 7.70 | 81.78 | <0.01** | 2.75 |
| Within | 6.03 | 64.00 | 0.09 |  |  |  |
| **Total** | **68.89** | **71.00** |  |  |  |  |
|  |  |  |  |  |  |  |
| **4D** |  |  |  |  |  |  |
| *Source of Variation* | *SS* | *df* | *MS* | *F* | *P-value* | *F crit* |
| Substrate | 64.47 | 1.00 | 64.47 | 160.50 | <0.01** | 3.99 |
| Genotype | 239.16 | 3.00 | 79.72 | 198.47 | <0.01** | 2.75 |
| Interaction | 187.21 | 3.00 | 62.40 | 155.36 | <0.01** | 2.75 |
| Within | 25.71 | 64.00 | 0.40 |  |  |  |
| **Total** | **516.55** | **71.00** |  |  |  |  |
|  |  |  |  |  |  |  |

| **4E** |  |  |  |  |  |  |
| --- | --- | --- | --- | --- | --- | --- |
| *Source of Variation* | *SS* | *df* | *MS* | *F* | *P-value* | *F crit* |
| Substrate | 0.14 | 1.00 | 0.14 | 4.36 | 0.04* | 3.99 |
| Genotype | 0.68 | 3.00 | 0.23 | 6.92 | <0.01** | 2.75 |
| Interaction | 0.46 | 3.00 | 0.15 | 4.72 | <0.01** | 2.75 |
| Within | 2.08 | 64.00 | 0.03 |  |  |  |
| **Total** | **3.36** | **71.00** |  |  |  |  |
|  |  |  |  |  |  |  |
| **4F** |  |  |  |  |  |  |
| *Source of Variation* | *SS* | *df* | *MS* | *F* | *P-value* | *F crit* |
| Substrate | 6.51 | 1.00 | 6.51 | 78.27 | <0.01** | 3.99 |
| Genotype | 26.78 | 3.00 | 8.93 | 107.39 | <0.01** | 2.75 |
| Interaction | 20.84 | 3.00 | 6.95 | 83.59 | <0.01** | 2.75 |
| Within | 5.32 | 64.00 | 0.08 |  |  |  |
| **Total** | **59.44** | **71.00** |  |  |  |  |
|  |  |  |  |  |  |  |
| **4G** |  |  |  |  |  |  |
| *Source of Variation* | *SS* | *df* | *MS* | *F* | *P-value* | *F crit* |
| Substrate | 4.79 | 1.00 | 4.79 | 68.76 | <0.01** | 3.99 |
| Genotype | 18.91 | 3.00 | 6.30 | 90.45 | <0.01** | 2.75 |
| Interaction | 13.78 | 3.00 | 4.59 | 65.91 | <0.01** | 2.75 |
| Within | 4.46 | 64.00 | 0.07 |  |  |  |
| **Total** | **41.95** | **71.00** |  |  |  |  |
|  |  |  |  |  |  |  |
| **4H** |  |  |  |  |  |  |
| *Source of Variation* | *SS* | *df* | *MS* | *F* | *P-value* | *F crit* |
| Substrate | 27.01 | 1.00 | 27.01 | 112.09 | <0.01** | 3.99 |
| Genotype | 104.61 | 3.00 | 34.87 | 144.71 | <0.01** | 2.75 |
| Interaction | 78.89 | 3.00 | 26.30 | 109.13 | <0.01** | 2.75 |
| Within | 15.42 | 64.00 | 0.24 |  |  |  |
| **Total** | **225.94** | **71.00** |  |  |  |  |
|  |  |  |  |  |  |  |

| **4I** |  |  |  |  |  |  |
| --- | --- | --- | --- | --- | --- | --- |
| *Source of Variation* | *SS* | *df* | *MS* | *F* | *P-value* | *F crit* |
| Substrate | 21.33 | 1.00 | 21.33 | 129.06 | <0.01** | 3.99 |
| Genotype | 85.52 | 3.00 | 28.51 | 172.53 | <0.01** | 2.75 |
| Interaction | 66.28 | 3.00 | 22.09 | 133.71 | <0.01** | 2.75 |
| Within | 10.58 | 64.00 | 0.17 |  |  |  |
| **Total** | **183.71** | **71.00** |  |  |  |  |
|  |  |  |  |  |  |  |
| **4J** |  |  |  |  |  |  |
| *Source of Variation* | *SS* | *df* | *MS* | *F* | *P-value* | *F crit* |
| Substrate | 3.56 | 1.00 | 3.56 | 45.38 | <0.01** | 3.99 |
| Genotype | 15.10 | 3.00 | 5.03 | 64.14 | <0.01** | 2.75 |
| Interaction | 11.36 | 3.00 | 3.79 | 48.26 | <0.01** | 2.75 |
| Within | 5.02 | 64.00 | 0.08 |  |  |  |
| **Total** | **35.04** | **71.00** |  |  |  |  |
|  |  |  |  |  |  |  |
| **4K** |  |  |  |  |  |  |
| *Source of Variation* | *SS* | *df* | *MS* | *F* | *P-value* | *F crit* |
| Substrate | 3.53 | 1.00 | 3.53 | 53.90 | <0.01** | 3.99 |
| Genotype | 15.76 | 3.00 | 5.25 | 80.11 | <0.01** | 2.75 |
| Interaction | 11.60 | 3.00 | 3.87 | 58.97 | <0.01** | 2.75 |
| Within | 4.20 | 64.00 | 0.07 |  |  |  |
| **Total** | **35.08** | **71.00** |  |  |  |  |
|  |  |  |  |  |  |  |
| **4L** |  |  |  |  |  |  |
| *Source of Variation* | *SS* | *df* | *MS* | *F* | *P-value* | *F crit* |
| Substrate | 21.95 | 1.00 | 21.95 | 124.30 | <0.01** | 3.99 |
| Genotype | 82.86 | 3.00 | 27.62 | 156.45 | <0.01** | 2.75 |
| Interaction | 65.52 | 3.00 | 21.84 | 123.70 | <0.01** | 2.75 |
| Within | 11.30 | 64.00 | 0.18 |  |  |  |
| **Total** | **181.62** | **71.00** |  |  |  |  |
|  |  |  |  |  |  |  |

| **4M** |  |  |  |  |  |  |
| --- | --- | --- | --- | --- | --- | --- |
| *Source of Variation* | *SS* | *df* | *MS* | *F* | *P-value* | *F crit* |
| Substrate | 2.01 | 1.00 | 2.01 | 27.74 | <0.01** | 3.99 |
| Genotype | 9.34 | 3.00 | 3.11 | 43.00 | <0.01** | 2.75 |
| Interaction | 7.84 | 3.00 | 2.61 | 36.11 | <0.01** | 2.75 |
| Within | 4.63 | 64.00 | 0.07 |  |  |  |
| **Total** | **23.82** | **71.00** |  |  |  |  |
|  |  |  |  |  |  |  |
| **5A** |  |  |  |  |  |  |
| *Source of Variation* | *SS* | *df* | *MS* | *F* | *P-value* | *F crit* |
| Substrate | 51.55 | 1.00 | 51.55 | 178.47 | <0.01** | 3.99 |
| Genotype | 181.51 | 3.00 | 60.50 | 209.48 | <0.01** | 2.75 |
| Interaction | 157.79 | 3.00 | 52.60 | 182.11 | <0.01** | 2.75 |
| Within | 18.48 | 64.00 | 0.29 |  |  |  |
| **Total** | **409.33** | **71.00** |  |  |  |  |
|  |  |  |  |  |  |  |
| **5B** |  |  |  |  |  |  |
| *Source of Variation* | *SS* | *df* | *MS* | *F* | *P-value* | *F crit* |
| Substrate | 40.29 | 1.00 | 40.29 | 137.01 | <0.01** | 3.99 |
| Genotype | 141.91 | 3.00 | 47.30 | 160.87 | <0.01** | 2.75 |
| Interaction | 123.25 | 3.00 | 41.08 | 139.72 | <0.01** | 2.75 |
| Within | 18.82 | 64.00 | 0.29 |  |  |  |
| **Total** | **324.27** | **71.00** |  |  |  |  |
|  |  |  |  |  |  |  |
| **5C** |  |  |  |  |  |  |
| *Source of Variation* | *SS* | *df* | *MS* | *F* | *P-value* | *F crit* |
| Substrate | 31.29 | 1.00 | 31.29 | 127.65 | <0.01** | 3.99 |
| Genotype | 110.25 | 3.00 | 36.75 | 149.92 | <0.01** | 2.75 |
| Interaction | 95.67 | 3.00 | 31.89 | 130.09 | <0.01** | 2.75 |
| Within | 15.69 | 64.00 | 0.25 |  |  |  |
| **Total** | **252.89** | **71.00** |  |  |  |  |
|  |  |  |  |  |  |  |

| **5D** |  |  |  |  |  |  |
| --- | --- | --- | --- | --- | --- | --- |
| *Source of Variation* | *SS* | *df* | *MS* | *F* | *P-value* | *F crit* |
| Substrate | 0.07 | 1.00 | 0.07 | 4.37 | 0.04* | 3.99 |
| Genotype | 2.69 | 3.00 | 0.90 | 55.19 | <0.01** | 2.75 |
| Interaction | 0.46 | 3.00 | 0.15 | 9.54 | <0.01** | 2.75 |
| Within | 1.04 | 64.00 | 0.02 |  |  |  |
| **Total** | **4.26** | **71.00** |  |  |  |  |
|  |  |  |  |  |  |  |
| **5E** |  |  |  |  |  |  |
| *Source of Variation* | *SS* | *df* | *MS* | *F* | *P-value* | *F crit* |
| Substrate | 0.06 | 1.00 | 0.06 | 2.92 | 0.09 | 3.99 |
| Genotype | 0.55 | 3.00 | 0.18 | 8.80 | <0.01** | 2.75 |
| Interaction | 0.28 | 3.00 | 0.09 | 4.40 | <0.01** | 2.75 |
| Within | 1.34 | 64.00 | 0.02 |  |  |  |
| **Total** | **2.23** | **71.00** |  |  |  |  |
|  |  |  |  |  |  |  |
| **5F** |  |  |  |  |  |  |
| *Source of Variation* | *SS* | *df* | *MS* | *F* | *P-value* | *F crit* |
| Substrate | 0.38 | 1.00 | 0.38 | 15.37 | <0.01** | 3.99 |
| Genotype | 2.06 | 3.00 | 0.69 | 28.03 | <0.01** | 2.75 |
| Interaction | 1.52 | 3.00 | 0.51 | 20.70 | <0.01** | 2.75 |
| Within | 1.57 | 64.00 | 0.02 |  |  |  |
| **Total** | **5.52** | **71.00** |  |  |  |  |
|  |  |  |  |  |  |  |
| **5G** |  |  |  |  |  |  |
| *Source of Variation* | *SS* | *df* | *MS* | *F* | *P-value* | *F crit* |
| Substrate | 0.51 | 1.00 | 0.51 | 58.07 | <0.01** | 3.99 |
| Genotype | 1.93 | 3.00 | 0.64 | 72.64 | <0.01** | 2.75 |
| Interaction | 1.46 | 3.00 | 0.49 | 55.17 | <0.01** | 2.75 |
| Within | 0.57 | 64.00 | 0.01 |  |  |  |
| **Total** | **4.47** | **71.00** |  |  |  |  |
| **P*<0.05, ***P*<0.01  †Following two-way ANOVA, Tukey’s post-hoc testing was used to determine significant differences between experimental groups. These results are indicated by **P*<0.05, ***P*<0.01 in the Figure panels. | | | | | | |

**SUPPLEMENTARY FIGURES**

**Figure S1. Network plots of the four gene co-expression modules associated with myelin substrate**

R-based bioinformatics analysis of published RNA-seq data (GEO accession no. GSE98974) from spinal cord-derived E12 mouse neural progenitor cells (E12 mNPCs) that were cultured on Poly-d-lysine control (PDL Ctrl), laminin (Lam), myelin (Mye), or Mye+Lam substrate [false discovery rate (FDR) < 0.1, n = 3 replicates/condition]. The CemiTools package in R was used to perform gene set enrichment analysis (GSEA)-based identification of gene co-expression modules. The network plots and associated hub genes of the four gene co-expression modules associated with Mye substrate – **(A)** M1, **(B)** M2, **(C)** M4, and **(D)** M6 -- are displayed.

**Figure S2. Reactome enrichment analysis for the four gene co-expression modules associated with myelin substrate**

The CemiTools package in R was used to perform a Reactome enrichment analysis for the four gene co-expression modules associated with Mye substrate: **(A)** M1, **(B)** M2, **(C)** M4, and **(D)** M6. The color saturation denotes the prediction confidence.

**Figure S3. Immunoblotting validation of CRISPR/Cas9-mediated *Mbp* knockout in the three mammalian NPC models.** Mbp-knockout (*Mbp^-/-^*) NPCs were constructed from wild-type (WT) NPCs using CRISPR/Cas9-based knockout of the *Mbp* gene. **(A)** WT and *Mbp^-/-^* spinal cord-derived E12 mouse neural progenitor cells (E12 mNPCs), **(B)** WT and *Mbp^-/-^* spinal cord-derived E14 rat neural progenitor cells (E14 rNPCs), and **(C)** D1 WT and *Mbp^-/-^* NPCs derived from human iPSCs (D1 hNPCs) were subjected to immunoblotting for Mbp protein expression after 48 hours of mouse or rat myelin (Mye) substrate culture. Gapdh used as the loading control.

**Figure S4. *Mbp* overexpression partially rescues myelin inhibition in mature NPCs.** **(A-E)** Wild-type (WT) spinal cord-derived E12 mouse neural progenitor cells (E12 mNPCs), **(F-J)** WT spinal cord-derived E14 rat neural progenitor cells (E14 rNPCs), or **(K-O)** D1 WT NPCs derived from human iPSCs (D1 hNPCs) were electroporated with either pcDNA3-Ctrl or pcDNA3-Mbp expression plasmid, then plated on Poly-d-lysine control (PDL Ctrl) (mNPCs, rNPCs) or laminin control (Lam Ctrl) substrate (D1 hNPCs). Following six-day maturation and trypsinization, NPCs were re-plated on mouse or rat myelin (Mye) substrate and analyzed for dendrite regeneration after a 24-hour period. **(A)** qPCR analysis of Mbp mRNA expression in transfected mNPCs. Gapdh used as the housekeeping control. **(B)** Total length of neurites per cell, **(C)** longest neurite per cell, **(D)** neurite branching for each cell, and **(E)** neurite initiation per cell. **(F)** qPCR analysis of Mbp mRNA expression in transfected rNPCs. Gapdh used as the housekeeping control. **(G)** Total length of neurites per cell, **(H)** longest neurite per cell, **(I)** neurite branching for each cell, and **(J)** neurite initiation per cell. **(K)** qPCR analysis of Mbp mRNA expression in transfected D1 hNPCs. Gapdh used as the housekeeping control. **(L)** Total length of neurites per cell, **(M)** longest neurite per cell, **(N)** neurite branching for each cell, and **(O)** neurite initiation per cell. All panels report means ± standard deviations (SDs). *n* = 3 embryos/genotype × 3 wells/embryo. **P*<0.05, ***P*<0.01 [**(A, F, K)** Student’s t-test; **(B-E, G-J, L-O)** two-way ANOVA, post-hoc Tukey’s test].

**Figure S1**

**
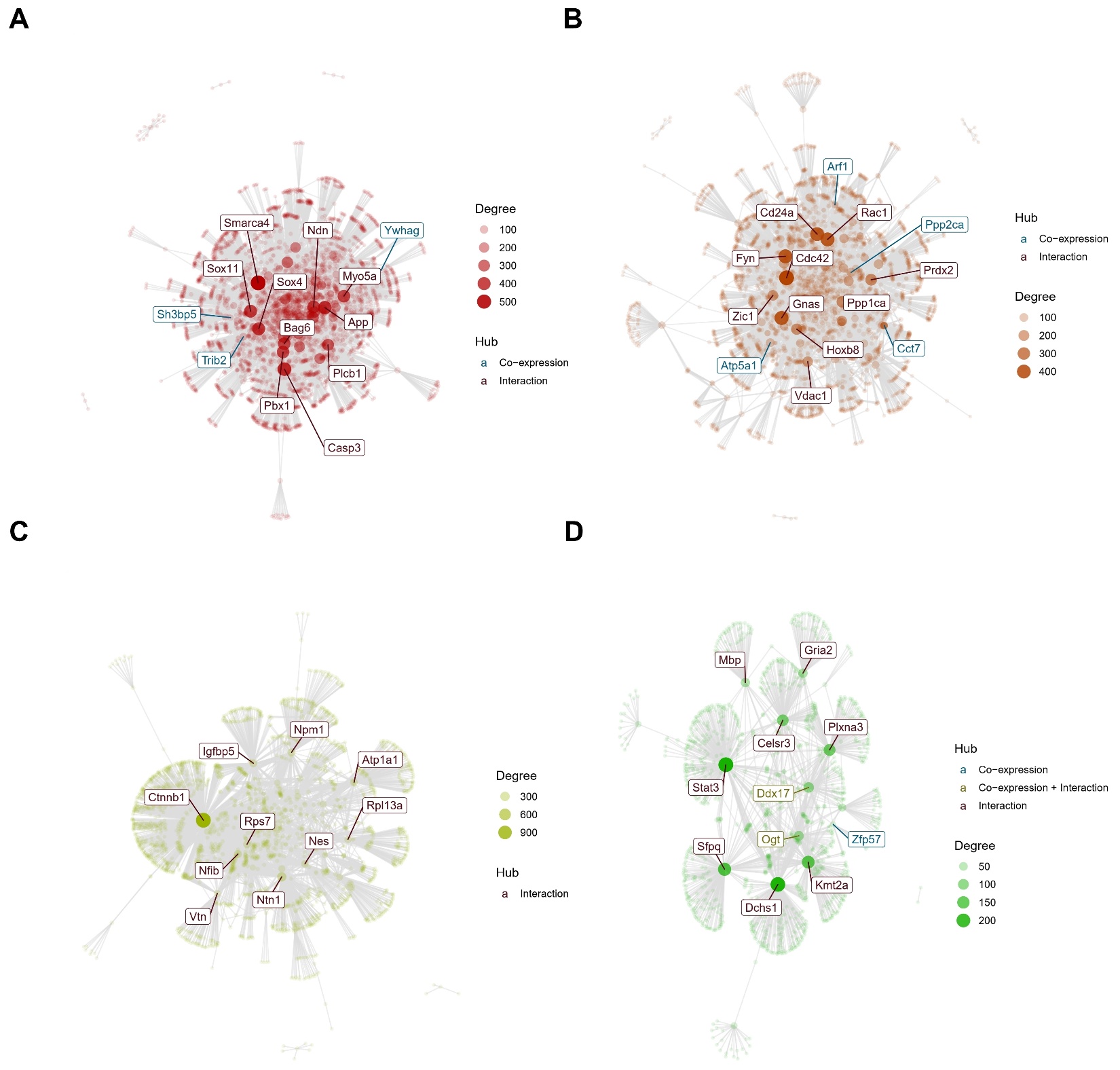
**

**Figure S4**

**
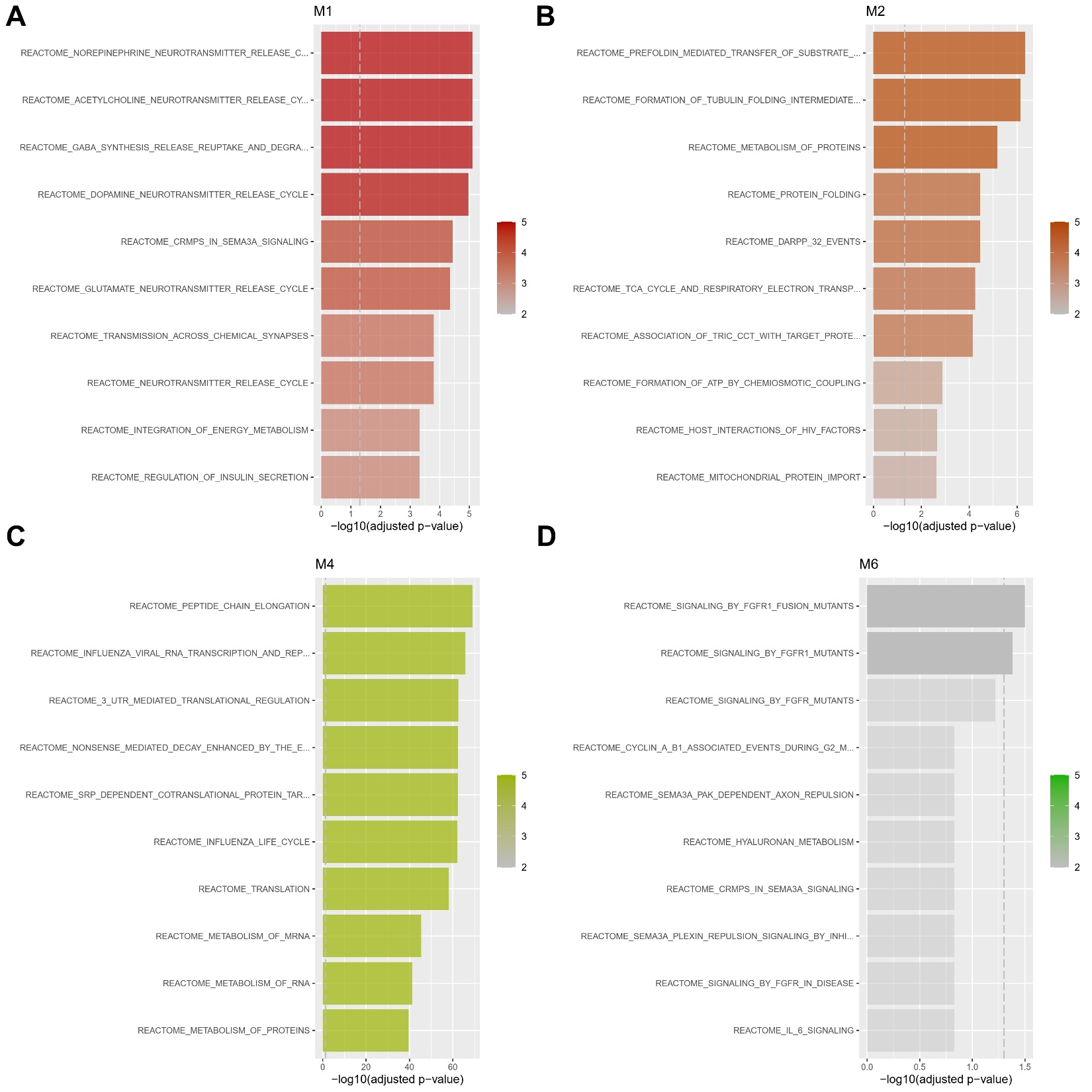
**

**Figure S3**

**
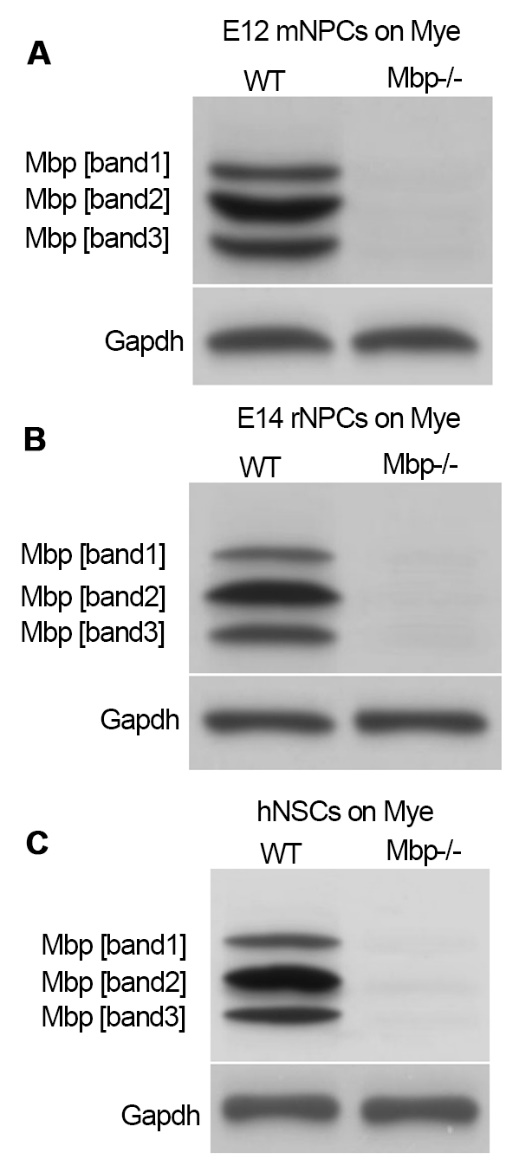
**

**Figure S4**

**
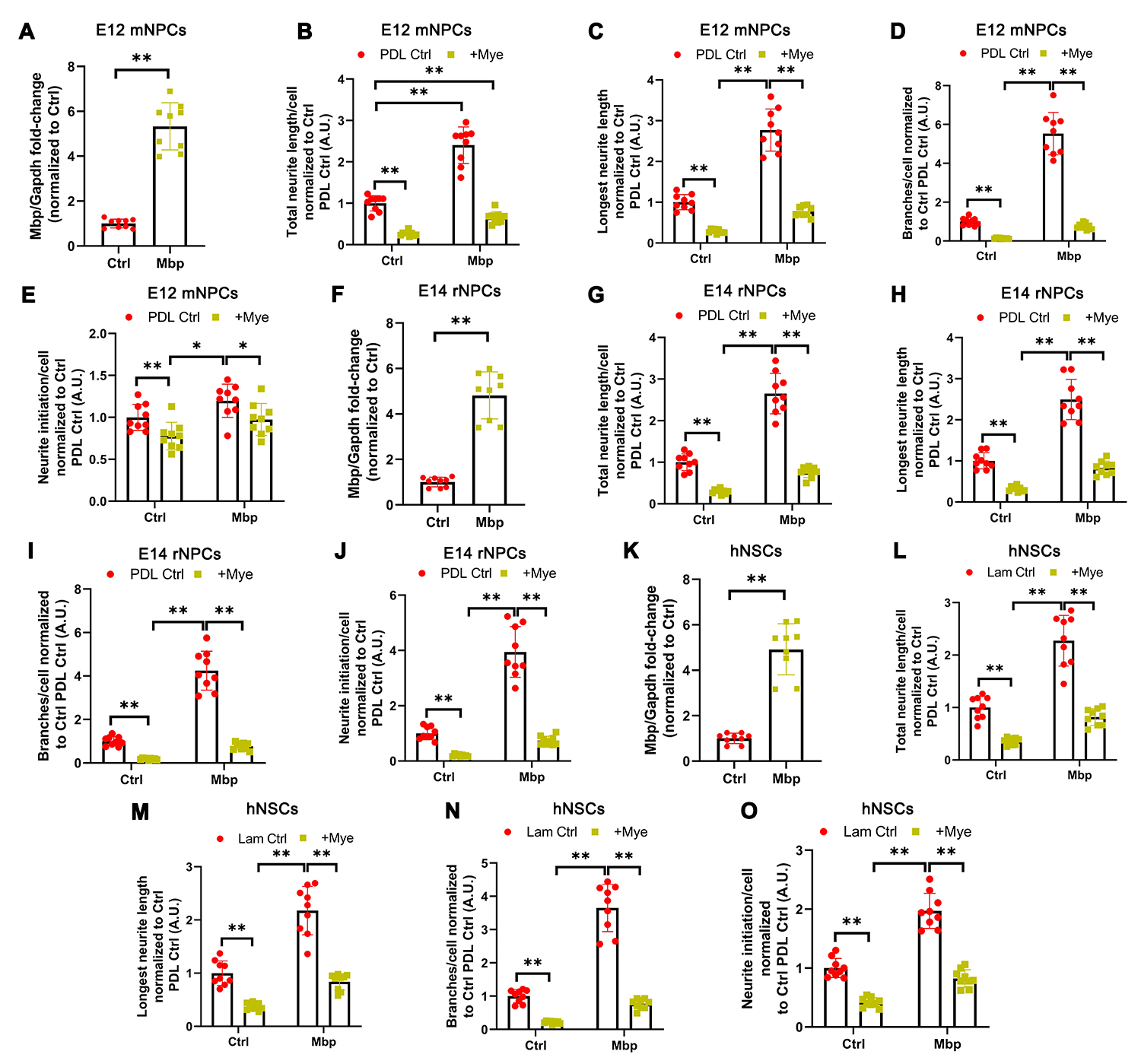
**

**REFERENCES FOR SUPPLEMENTARY INFORMATION**

1. Poplawski GH, Lie R, Hunt M, Kumamaru H, Kawaguchi R, Lu P, et al. Adult rat myelin enhances axonal outgrowth from neural stem cells. Science Translational Medicine. 2018;10(442):eaal2563.

2. Ille A, Lamont H, Ruvio S, Bernosky‐Smith K. CRISPR/Cas9‐Mediated Rescue of Shiverer Mice (MBPShi) via AAV9 Delivery across the Blood‐Brain Barrier. The FASEB Journal. 2019;33(S1):662.5-.5.

3. Anderson KR, Haeussler M, Watanabe C, Janakiraman V, Lund J, Modrusan Z, et al. CRISPR off-target analysis in genetically engineered rats and mice. Nature methods. 2018;15(7):512-4.

4. Lee AW, Hengstler H, Schwald K, Berriel-Diaz M, Loreth D, Kirsch M, et al. Functional inactivation of the genome-wide association study obesity gene neuronal growth regulator 1 in mice causes a body mass phenotype. PloS one. 2012;7(7):e41537.

5. Larocca JN, Norton WT. Isolation of myelin. Current Protocols in Cell Biology. 2006;33(1):3.25. 1-3.. 19.

6. Kadoya K, Lu P, Nguyen K, Lee-Kubli C, Kumamaru H, Yao L, et al. Spinal cord reconstitution with homologous neural grafts enables robust corticospinal regeneration. Nature medicine. 2016;22(5):479.

7. Geng B-c, Choi K-H, Wang S-z, Chen P, Dong N-g, Ko J-K, et al. A simple, quick, and efficient CRISPR/Cas9 genome editing method for human induced pluripotent stem cells. Acta Pharmacologica Sinica. 2020:1-6.

8. Israel MA, Yuan SH, Bardy C, Reyna SM, Mu Y, Herrera C, et al. Probing sporadic and familial Alzheimer’s disease using induced pluripotent stem cells. Nature. 2012;482(7384):216-20.

9. Li Y, Lazar MA. Differential gene regulation by PPARγ agonist and constitutively active PPARγ2. Molecular Endocrinology. 2002;16(5):1040-8.

10. McCall J, Nicholson L, Weidner N, Blesch A. Optimization of adult sensory neuron electroporation to study mechanisms of neurite growth. Frontiers in molecular neuroscience. 2012;5:11.

11. Perron JC, Bixby JL. Distinct neurite outgrowth signaling pathways converge on ERK activation. Molecular and Cellular Neuroscience. 1999;13(5):362-78.

12. Yu Z, Cheng C, Liu Y, Liu N, Lo EH, Wang X. Neuroglobin promotes neurogenesis through Wnt signaling pathway. Cell death & disease. 2018;9(10):1-12.

13. Kourgiantaki A, Tzeranis DS, Karali K, Georgelou K, Bampoula E, Psilodimitrakopoulos S, et al. Neural stem cell delivery via porous collagen scaffolds promotes neuronal differentiation and locomotion recovery in spinal cord injury. NPJ Regenerative medicine. 2020;5(1):1-14.
